# Explaining Twitter’s inability to effectively moderate content during the COVID-19 pandemic

**DOI:** 10.1038/s41598-025-20033-6

**Published:** 2025-10-15

**Authors:** David A. Broniatowski, Wei Zhong, Joseph R. Simons, Amelia M. Jamison, Mark Dredze, Lorien C. Abroms

**Affiliations:** 1 Department of Engineering Management and Systems Engineering, The George Washington University, Washington, DC 20052 USA; 2 Center for Social Media and Politics, New York University, New York, NY 10012 USA; 3 Office of the Assistant Secretary for Financial Resources, United States Department of Health and Human Services, Washington, DC 20201 USA; 4 Department of Civil and Systems Engineering, Johns Hopkins University, Baltimore, MD 21218 USA; 5 Department of Computer Science, Johns Hopkins University, Baltimore, MD 21218 USA; 6 Department of Prevention and Community Health, The George Washington University, Washington, DC 20037 USA

**Keywords:** Information technology, Computational science

## Abstract

Social media platforms routinely face pressure to restrict harmful content while protecting free speech; however, prior theory suggests that platform design might undermine the efficacy of content moderation. During the COVID-19 pandemic, major social media platforms removed content that violated their medical misinformation policies. Although controversial, it is widely assumed that these interventions, such as deplatforming and content removal, are efficacious; however, this claim has not been evaluated based on evidence. We therefore evaluated the efficacy of Twitter’s attempts to curtail vaccine misinformation during the COVID-19 pandemic. We found that several clusters of vaccine skeptical accounts generated a larger share of tweets about vaccines, increased in virality, and continued to spread low-quality and likely misinformative content despite Twitter’s sustained removal of content and accounts — both when compared to prior trends and to corresponding clusters of pro-vaccine accounts. Several of these accounts were subject to a contemporaneous moderation action, in which Twitter removed 70,000 accounts on January 8, 2021. Although this action reduced activity among targeted accounts, virality increased and information quality decreased among both these accounts and those sharing similar political affiliations, calling into question the efficacy of these removals. Novel platforms that share Twitter’s architecture may therefore face similar moderation challenges.

## Introduction

Social media platforms shape the flow of information throughout society. These platforms facilitate easy access to overwhelming amounts of information^1,2^, and can interfere with users’ ability to discern truth from falsehood^3,4^. Thus, misinformation on these platforms has become a well-documented and longstanding concern, with implications for issues as varied as science, disaster response, public health, and democratic processes^5–15^. Moreover, there is now concrete evidence associating online collective behavior with offline harmful behavior^16–18^. It is therefore increasingly important to ensure that people seeking quality information can access it.

Several segments of society have expressed dissatisfaction with how social media platforms manage the flow of information online^19,20^. For example, pressure has come from advertisers who do not want their products associated with harmful content^21^. More recently, governments have leveled criminal charges at social media platform executives and threatened corporate entities with sanctions for not taking action against harmful content^22,23^. Finally, some evidence suggests that citizens prefer when platforms remove misinformation^24^. However, when several mainstream platforms did so during the COVID-19 pandemic, they were subject to legal threats from users who felt that they, or their content, were removed unfairly^25^.

These actions implicitly assume that platforms’ efforts to stem misinformation can be successful, subject only to the desire of platform leadership to carry them out. However, this claim has not yet been scientifically evaluated. A limited number of studies suggest that some interventions might be efficacious on some platforms; however, results are mixed^26–28,28–38^. The most relevant example from prior work to our inquiry provides some evidence that Twitter (now called X [Throughout the rest of this paper, we refer to the platform as Twitter since that was its name during our period of study.]) reduced misinformation on the platform by suddenly removing 70,000 accounts — including the account of the sitting President of the United States — following political violence at the US Capitol on January 6, 2021^36^. These findings are directly relevant to our inquiry because Twitter was one of the world’s largest and most widely used social media platforms. However, a counterexample is found in prior work showing that Facebook’s attempts to reduce vaccine misinformation during the height of the COVID-19 pandemic had limited efficacy^31^. These two studies face complementary limitations: McCabe et al.^36^ did not examine multiple communities, nor did they examine the long term efficacy of Twitter’s intervention, whereas Broniatowski et al.^31^ did not disentangle the effects of content removal from those of deplatforming because Facebook implemented these remedies roughly simultaneously.

We attempted to explain these contradictory findings by drawing upon prior evidence-based theory to simulate how social media platform architectures facilitate or inhibit such attempts (see Supplementary Information). Crucially, Facebook and Twitter possess different architectures. According to our theory, some platform architectures are more *flexible* than others because they enable users to easily establish new paths to interdicted content, thus facilitating resistance to content moderation efforts. Facebook, a layered hierarchy, is hypothesized to be only moderately flexible while Twitter, an ahierarchical network, is hypothesized to be more flexible^39^. Thus, our theory, and corresponding simulation results (see Supplementary Information & Tables S1-S6) suggest that – all else being equal – content moderation policies should be less efficacious on Twitter than on Facebook. Interpreting our findings in light of a theory of architecture provides a unique source of novelty to this study, moving beyond prior work which has reported the efficacy of isolated interventions without explaining why they might have succeeded or failed. Indeed, if this theory is correct, it has implications for the expected success of moderation efforts on new platforms that share architectural features with legacy platforms. For example, the membership of Mastodon and Bluesky^40,41^, platforms whose user interfaces strongly resemble Twitter’s, increased to over 20 million users in early November, 2024^42^. These new platforms offer content moderation; for example, Bluesky offers users the ability to opt in to moderation and labeling services provided by the platform. However, if our theory is correct, Twitter’s experiences with content moderation may predict the success or failure of these platforms’ efforts.

In this paper, we use quasi-experimental designs to evaluate the effects of Twitter’s content removal policies on the amount, virality, quality, and topics of vaccine-related content on Twitter. On December 16, 2020, Twitter announced its intention to remove vaccine misinformation as part of its medical misinformation control efforts [https://blog.x.com/en_us/topics/company/2020/covid19-vaccine]. Then, on March 1, 2021, Twitter implemented a “five strikes” policy under which accounts that repeatedly posted medical misinformation would receive several warnings of escalating severity, culminating in their deplatforming if their behavior persisted [https://blog.x.com/en_us/topics/company/2021/updates-to-our-work-on-covid-19-vaccine-misinformation]. One of our novel contributions is that we simultaneously evaluate and disentangle the effects of content removal and deplatforming in two ways: first, by taking advantage of the fact that changes to Twitter’s content removal and account suspension policies were made at distinct points in time and, second, by including separate predictors for content and account removal volumes in a Least Squares Dummy Variable (LSDV) analysis, thus addressing a limitation of prior work.

Twitter’s decision to stop enforcing its medical misinformation policies on November 23, 2022, was widely criticized^43^, under the assumption that they successfully prohibited misinformation spread. Using a dataset of more than 400 million tweets collected from over 6.7 million accounts between February 6, 2020, and December 17, 2022, we evaluate the platform-level effects of these policies.

### Twitter’s architecture facilitates information spread between communities

**Fig. 1:**
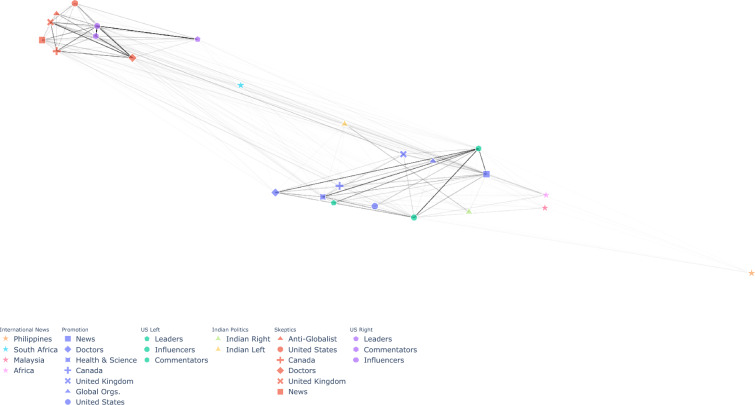
Network diagram showing information flows between the 25 communities identified. Edge weights indicate retweet frequency. Vaccine skeptical communities (red) are tightly entangled with the US Political Right (purple), whereas vaccine promoting communities (blue) are entangled with the US Political Left (green).

We identified 25 communities in our dataset using a community detection algorithm applied to a retweet network made up of all users that were connected to at least three other users (see Methods). To illustrate how this structure can facilitate flexibility consider that Twitter does not impose any restrictions on which accounts may share (retweet) content from other accounts. Thus, its architecture, when combined with popularity-based recommendation algorithms, facilitates a positive feedback loop, whereby popular accounts are more likely to be retweeted, further increasing their popularity (the “rich get richer” dynamic). As a result, Twitter’s structure has been approximated by a scale-free network^44^, which implies that communities of accounts are themselves clustered into communities that are likely to share information within a broader ecosystem. Furthermore, the scale-free nature of Twitter implies that these communities are likely to be entangled with a broader vaccine skeptical ecosystem. The network diagram shown in Figure 1 demonstrates that the identified community of prominent US Vaccine Skeptics accounts is embedded within a larger ecosystem comprising several different vaccine skeptical — but also political — communities that frequently retweet one another’s content. These communities are distinct from a separate set of communities sharing messages that generally promoted vaccines, which were entangled with accounts prominent in the US left-wing political discourse. This structure demonstrates that attempts to moderate content in one community could be easily circumvented by accounts in nearby communities.

Our dataset spans almost three full years, allowing us to evaluate the long-run efficacy of Twitter’s interventions, thus addressing another limitation of prior work. We compare the efficacy of Twitter’s interventions across all identified communities of Twitter accounts, including communities formed around leading vaccine skeptics in the United States, but also other communities that both promoted and were skeptical of vaccines. Comparing across these communities is another source of novelty for our work. Specifically, we ask whether Twitter’s policies

1. reduced tweets about vaccines from vaccine skeptical accounts;
2. reduced the virality of these tweets;
3. decreased the likelihood that content violating Twitter’s medical misinformation policies was shared by these accounts.

Given the observed clustering between accounts discussing vaccination and accounts expressing political sentiments, and prior work examining the impacts of Twitter’s deplatforming efforts^36^, we also examine the effects of these actions on our dependent variables on communities of accounts sharing the same political affiliation as deplatformed accounts, including those in the same community as the deplatformed US President.

Failure to answer these questions systematically will inhibit society’s ability to ensure that citizens can access much needed information, undermining their ability to make informed decisions and leaving them vulnerable to mass manipulation, while also encouraging expenditure of significant resources on policies that might be ineffective or even counterproductive. Given the high-stakes nature of enforcing such policies, our study provides a crucial scientific assessment of whether, and which types, of content moderation remedies are likely to be effective.

## Results

We tested the hypothesis that Twitter’s interventions did not effectively reduce the spread of vaccine misinformation — as defined by their medical misinformation policies — on the platform. First, we evaluated whether the introduction of new policies was associated with a reduction in content, virality, and vaccine skeptical content in vaccine skeptical Twitter communities as compared to vaccine promoting communities on the platform. We used a daily comparative interrupted time series (CITS) design to identify the effect of Twitter’s policies in 25 identified communities of Twitter accounts. In a press release dated December 16, 2020, Twitter announced that it would begin removing vaccine misinformation “next week” – a fact that was confirmed by Twitter’s transparency data, which show a surge in content removals in January 2021. We therefore measure post-intervention content sharing as beginning December 20, 2020 – the beginning of the week following Twitter’s announcement. In addition, in a press release dated March 1, 2021, Twitter announced the introduction of its “five strikes” policy. We therefore use this date to index the beginning of a period during which Twitter removed several prominent accounts that repeatedly spread content violating Twitter’s medical misinformation policies.

Interpreting the magnitude of the CITS estimates as causal effects of Twitter’s interventions requires assuming that other roughly contemporaneous events, such as the worldwide rollout of COVID vaccines or the January 6th violence at the US Capitol and Twitter’s subsequent deplatforming of 70,000 accounts, had equal effects across all control and treatment groups. The CITS design has a causal interpretation, yet it is only valid to the extent that these assumptions are correct. We therefore deliberately frame our results in “non-causal” language. If the policy was efficacious, we would expect to see a decrease in misinformative content, and one could, in principle, interpret this as causal evidence for the policy’s efficacy. In contrast, although the absence of an effect is statistically inconclusive, an effect in the *opposite direction* (i.e., an increase in misinformative content following a policy designed to decrease it) is a clear indication that the policy failed to decrease misinformation relative to comparators. We therefore examine our results across multiple different controls as a robustness check.

### Effects on prominent US skeptics

Prior work often begins by examining an isolated community of Twitter accounts. Consider the community comprising the most prominent vaccine skeptics in the United States. Among the accounts in this community are half of the accounts that the Center for Countering Digital Hate dubbed the “Disinformation Dozen” [https://counterhate.com/research/the-disinformation-dozen/]. Several news sources and prominent figures called for these accounts to be deplatformed^45^; therefore, they are likely to have been among the most salient targets of Twitter’s interventions. To gain initial insight into whether Twitter’s policies were associated with relative changes to these accounts in isolation, we compared their activity to those of a second community of accounts operated by several U.S. public health agencies (“US Vaccine Promotion”). We conducted a CITS analysis comparing these two communities by fitting Seasonal Autoregressive Integrated Moving Average models with eXternal regressors (SARIMAX) to data from before Twitter’s content removal and “five strikes” policies were implemented. Next, we used these SARIMAX models to forecast counterfactuals under the assumption that Twitter’s policies had not been implemented, comparing these forecasts to actual data (see Figure S1). Dependent variables included the proportions of tweets across these communities, virality (retweets per follower) of those tweets, daily average information quality, and the daily proportions of tweets discussing topics that either violated Twitter’s medical misinformation policies, commented on vaccines in a manner that expressed vaccine skepticism without necessarily violating these policies, or promoted vaccination.

#### Prominent US skeptics produced proportionally less content

**Fig. 2:**
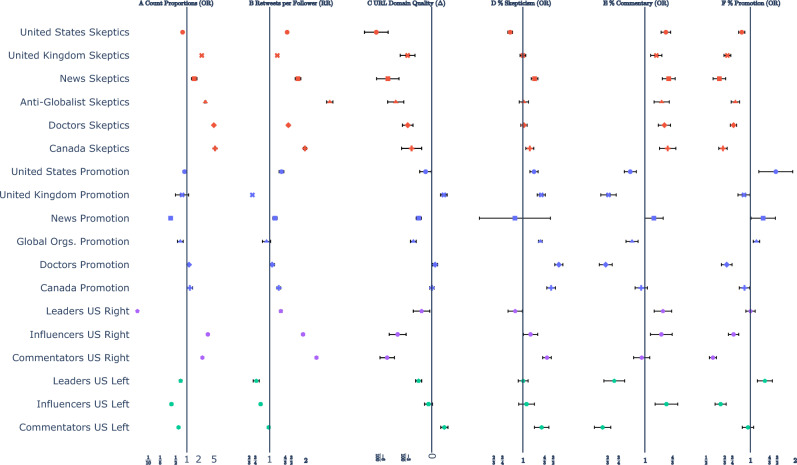
During the “five strikes” phase, CITS results show (**A**) Increases in content proportion for each vaccine skeptical community relative both to counterfactual trends based on prior data and their respective comparators for all but the Prominent US Skeptics and US Right Leaders communities; (**B**) Increases in virality (Retweets per follower) for all Vaccine Skeptical and US Right communities; (**C**) Decreases in URL Domain Quality for all Vaccine Skeptical and US Right communities (with the exception of US Right Leaders, which decreased significantly compared to counterfactual trends but not relative to US Left Leaders). (**D**) Vaccine promotion communities, and US Left Commentators largely increased discussion of topics that expressed vaccine skepticism, whereas Vaccine Skeptical communities either did not change or increased discussion of these topics relative to counterfactual trends based on prior data, with the exception of the Prominent US Skeptics community. We also observed (**E**) increased discussion of Commentary topics and (**F**) decreased discussion of vaccine promotion topics among all Vaccine Skeptical communities.

During both the content removal, *OR = 0.33, 95% CI: 0.28–0.38*, $p<0.001$, and “five strikes”, *OR = 0.78, 95% CI: 0.75–0.82*, $p<0.001$, periods, the observed proportion of tweets from Prominent US Skeptics was significantly lower than the counterfactual predictions generated by the SARIMAX model based on prior data (detailed statistical results are shown in Table S7). Content also decreased when compared to corresponding decreases in content proportions from US Vaccine Promotion, *OR = 0.37, 95% CI: 0.24 — 0.58, *p<*0.001* and *OR = 0.89, 95% CI: 0.80 — 0.99, p=0.02*, respectively (see Figure 2A, first row).

#### Virality of prominent US skeptics’ tweets increased

During the content removal phase, virality of content from US Vaccine Skeptics’ accounts decreased only slightly relative to counterfactual projections derived from the SARIMAX model, *RR = 0.93, 95% CI: 0.87 — 0.99, *$p=0.01$ but not relative to decreases in the US Vaccine Promotion (i.e., US Health Agencies) community, *RR = 1.05, 95% CI: 0.86 — 1.28, 0.32*. However, this decrease was smaller than an increase in virality during the “five strikes” phase both compared to trends derived from prior data (see Table S7), *RR = 1.40, 95% CI: 1.37 — 1.43, *$p<0.001$ and US Vaccine Promotion, *RR = 1.12, 95% CI: 1.06 — 1.18, *$p<0.001$ (see Figure 2B, first row).

#### Prominent US skeptics’ information quality initially increased, but then decreased

The quality of URL domains shared during the content removal phase increased relative to trends derived from prior data, $\Delta$ = *0.03, 95% CI: 0.00 — 0.06*, $p=0.03$ and relative to US Vaccine Promotion, $\Delta$ = *0.04, 95% CI: 0.01 — 0.08*, $p=0.004$ see Table S7). However, these gains were overcome by larger losses during the “five strikes” phase where URL domain quality decreased relative to trends derived from prior data, $\Delta$ =*−0.07, 95% CI: −0.08 — −0.05*, $p<0.001$, and US Vaccine Promotion, $\Delta$ =*−0.06, 95% CI: −0.07 — −0.04*, $p<0.001$ (see Figure 2C, first row).

#### Prominent US skeptics may have exhibited reactance

During the content removal phase, the proportion of vaccine skeptical content increased relative to counterfactual trends derived from prior data, *OR = 1.49, 95% CI: 1.35 — 1.63, *$p<0.001$ and relative to US Vaccine Promotion, *OR = 2.71, 95% CI: 2.26 — 3.24, *$p<0.001$, see Table S7. However, these gains were partially reversed during the “five strikes” phase by decreases relative to trends derived from prior data, *OR = 0.84, 95% CI: 0.81 — 0.86, *$p<0.001$, and US Vaccine Promotion, *OR = 0.72, 95% CI: 0.68 — 0.77, *$p<0.001$ (see Figure 2D, first row). Concurrently, discussion of commentary topics, including concerns regarding violations of civil liberties, morality, and politics, increased relative to trends derived from prior data, *OR = 1.40, 95% CI: 1.37 — 1.43, *$p<0.001$, and US Vaccine Promotion, *OR = 1.12, 95% CI: 1.06 — 1.18*, $p<0.001$ (see Figure 2E, first row).

### Effects on other vaccine skeptical accounts

The effects of Twitter’s policies on US Vaccine Skeptical accounts, when analyzed in isolation, appear to be similar to those observed after Facebook implemented its own anti-vaccine content removal measures^31^. However, these accounts were embedded in a larger ecosystem of vaccine skeptical accounts. We therefore next examined the efficacy of Twitter’s policies on the platform as a whole.

**Fig. 3:**
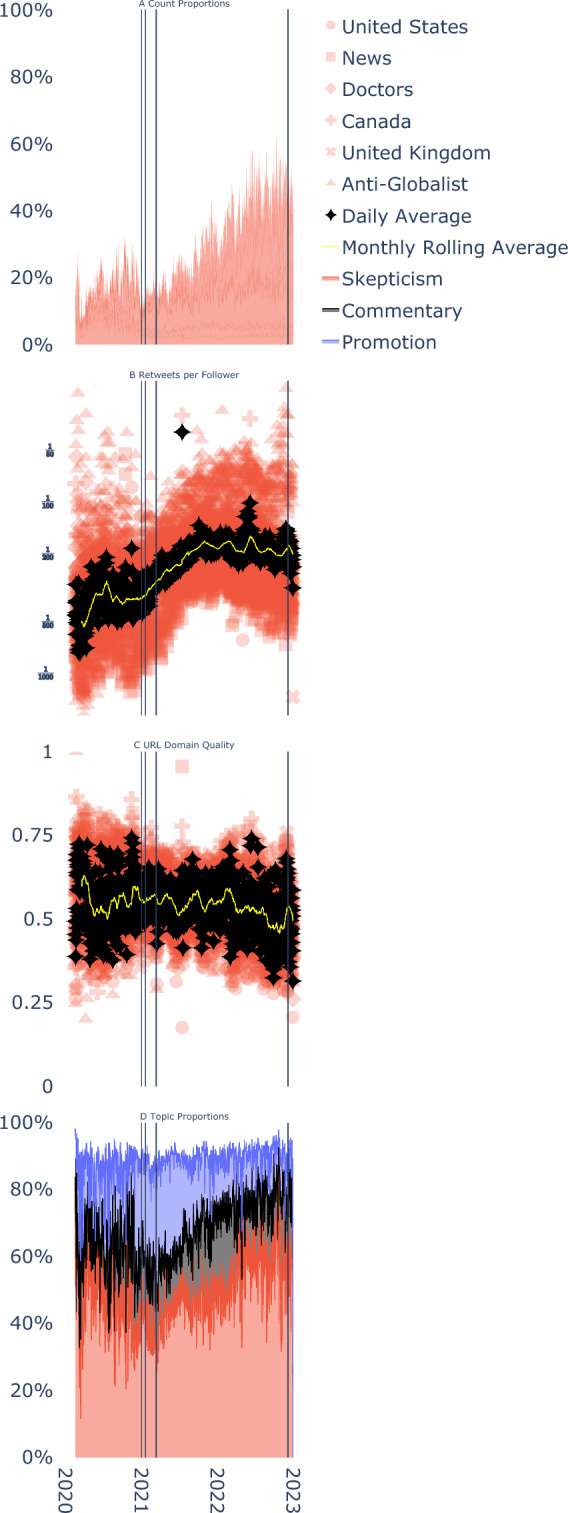
(**A**) daily proportion of tweets about COVID vaccines generated by all vaccine skeptical accounts. Overall proportion of content generated by vaccine skeptical communities increased to over 60% of sampled tweets. (**B**) daily average $\log$ retweets per follower for tweets generated by vaccine skeptical communities. A 30-day rolling average, shown in yellow, demonstrates a steady increase during the “five strikes” phase. (**C**) daily average URL domain quality for tweets generated by vaccine skeptical communities. A 30-day rolling average, shown in yellow, demonstrates a steady decrease during the “five strikes” phase. Tweets from US Vaccine Skeptics tend to have the lowest quality URLs. (**D**) daily proportion of tweets that discuss topics: (red) expressing vaccine skepticism, (black) discussing civil liberties, morality, or political commentary, and (blue) promoting vaccination. Vertical lines indicate when Twitter’s content removal policy commenced, when Twitter deplatformed 70,000 accounts, when Twitter commenced its “five strikes” policy, and when Twitter’s medical misinformation policy was rescinded.

#### Other communities of Vaccine Skeptical Accounts Became More Active, More Viral, More Misinformative, and May Have Exhibited Reactance

Figure 3A shows that the proportion of content generated by vaccine skeptical communities steadily increased from roughly 25% of all tweets before Twitter implemented its content moderation policies, to roughly 60% of all tweets in our sample by the end of 2022. During this phase, virality of content generated by vaccine skeptical communities also increased by a factor of roughly 2.5, from approximately 1 retweet per 500 followers to 1 retweet per 200 followers (Figure 3B), whereas URL domain quality remained roughly constant and – by the end of the “five strikes period” – decreased (Figure 3C). Furthermore, tweets were more likely to contain content that expressed vaccine skepticism, and less likely to discuss vaccine promotion by the end of the “five strikes” period (Figure 3D).

Examining each community separately, although US Prominent US Skeptics, Vaccine Skeptical News sources, and Anti-Globalist accounts briefly decreased their relative activity the content removal phase relative both to SARIMAX-generated counterfactual projections and pro-vaccine comparators (Table S7), all communities—except for the Prominent US Skeptics—showed significant increases in activity during the “five strikes” phase relative to pro-vaccine comparators and counterfactual trends derived from prior data (Figure 2A). These increases were especially pronounced among Vaccine Skeptics in the Canada, *OR = 5.31, 95% CI: 5.04–5.60, *$p<0.001$, Doctors, *OR = 4.93, 95% CI: 4.78–5.08, *$p<0.001$, Anti-Globalist, *OR = 2.99, 95% CI: 2.88–3.10*, $p<0.001$, and News, *OR = 1.56, 95% CI: 1.33–1.84, *$p<0.001$ communities.

Virality, measured as retweets per follower, increased significantly among several communities during both policy phases. During the content removal phase, virality rose in the Vaccine Skeptical Canada and Doctors communities (see Table S7). In the “five strikes” phase, all skeptical communities showed statistically significant increases relative both to SARIMAX-generated counterfactuals based on prior trends and to pro-vaccine comparators (see Figure 2B).

Domain-level URL quality ratings improved significantly for Vaccine Skeptics during the content removal phase in the Canada, Doctors, and United Kingdom communities, relative to their pro-vaccine comparators (Table S7). However, these gains were reversed in the “five strikes” phase, when statistically significant declines in information quality occurred across all skeptical communities. Prominent US Skeptics exhibited the largest drop in information quality, $\Delta$ =*−0.07, 95% CI: −0.08 – −0.05*,$p<0.001$) (Figure 2C).

An analysis of topic content provides additional insight into how discourse evolved under Twitter’s enforcement regimes. During the content removal phase, the likelihood that accounts discussed topics expressing vaccine skepticism increased in all but the UK and and Canada communities, relative to pro-vaccine comparators. Specifically, vaccine-skeptical communities increased relative to comparators in the United States (*OR* = 2.71, 95% CI: 2.26–3.24, $p < 0.001$), among Doctors (*OR* = 1.37, 95% CI: 1.13–1.67, $p < 0.001$), and in the anti-globalist community *OR* = 1.57, 95% CI: 1.19–2.07, $p < 0.001$. Furthermore, during the “five strikes” period, we only observed a decrease in skeptical content proportions in the Prominent US Skeptics community, *OR = 0.84, 95% CI: 0.81–0.86, *$p<0.001$. Notably, vaccine promoting communities appear to have increased their discussion of skeptical content significantly more than corresponding vaccine skeptical communities for all but the Mainstream News community, suggesting that the discourse in pro-vaccine communities increasingly focused on countering vaccine skeptical narratives (see Figure 2D).

Concurrently, all vaccine-skeptical communities consistently discussed more commentary pertaining to civil liberties, morality, and politics than their pro-vaccine counterparts during the “five strikes” phase (see Figure 2E), suggesting that accounts in these communities increasingly framed vaccine refusal as a civil right^46^ with moderation efforts possibly triggering “psychological reactance” — a situation in which “when something threatens or eliminates people’s freedom of behavior, they experience... a motivational state that drives freedom restoration”^47^potentially galvanizing opposition^48^. By contrast, pro-vaccine messages were less often framed in this manner.

Finally, the share of vaccine promoting content decreased significantly among skeptical communities, especially during the “five strikes” phase (Figure 2F).

### Separate effects of content and account removals

The dearth of studies in this domain is due, in part, to the difficulty in obtaining systematic data regarding platforms’ activities and the fact that platforms’ policies are often implemented incrementally and, seemingly, arbitrarily^36^. The analysis above relies on techniques that are similar to those used in previous studies attempting to estimate causal effects by examining the effects of single massive deplatforming events^32,36^. In practice, such actions are rare because they expose platforms to significant risk. In contrast, incremental actions taken by social media platforms are much more commonplace. We therefore evaluated these incremental actions using Twitter’s own reports of platform-wide counts of monthly accounts suspended and pieces of content removed. To do so, we took advantage of transparency data provided by Twitter, which provides monthly counts of pieces of content and the number of accounts removed for July 2020 through September 2022. [These data are currently available at https://web.archive.org/web/20240923000645/https://transparency.x.com/en/reports/covid19#expand:2021-jul-dec:] In order to control for unmodeled heterogeneity in each community, and differences in their propensity to share misinformative content, we estimated a fixed effects model using the Least Squares Dummy Variable (LSDV) approach, with a dichotomous dummy for each Twitter community (see Materials and Methods; Figure S2 & Tables S8, S9, & S10). Furthermore, this model is not sensitive to one of the core assumptions of the CITS design — that treatment and control groups share confounders. Results that replicate between CITS and LSDV designs are therefore likely to be robust to violations of this assumption. Thus, the inclusion of multiple comparator communities and the LSDV robustness check allow us to draw meaningful conclusions across communities despite potentially heterogeneous responses. Finally, the LSDV model includes separate predictors for content and account removals, enabling us to estimate the distinct contributions of each type of moderation intervention. This analysis largely replicates our CITS findings (see Supplementary Information).

### Effect of removing large numbers of accounts

Despite the international scope of vaccine skepticism, Figure 1 shows that the vaccine skeptical communities in our sample are tightly clustered with communities representing the political right wing in the United States. On January 8, 2021, Twitter deplatformed 70,000 accounts following violence at the U.S. Capitol on January 6, 2021. Although not explicitly targeting vaccine misinformation, prior work suggests that this deplatforming action reduced the overall spread of misinformation on the platform^36^. The politicization of COVID-19 vaccine hesitancy is well documented^49–51^. We therefore examined whether this policy had a measurable effect on COVID vaccine misinformation in the other political communities in our sample.

#### Deplatforming reduced content proportions in targeted accounts

**Fig. 4:**
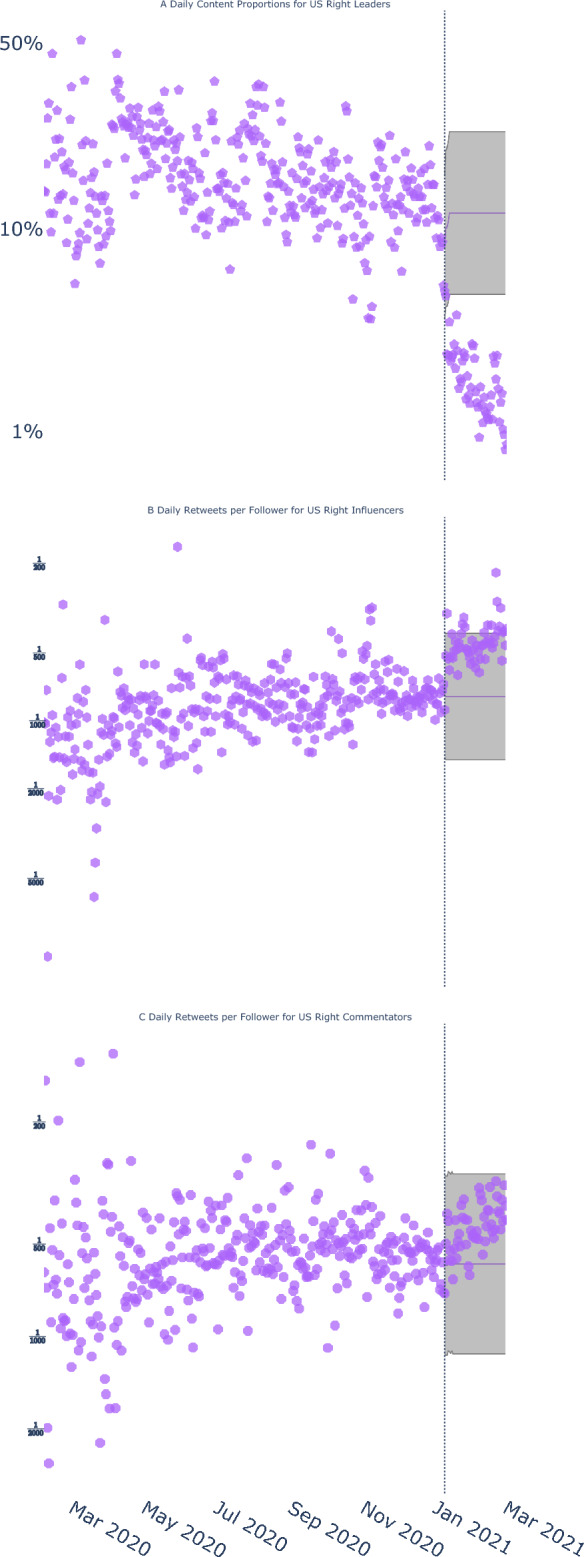
Despite the removal of a community of accounts containing followers of the sitting US President^36^, activity in other politically-aligned accounts seems to have increased. (**A**) Daily content proportions for tweets from the community containing the US President (US right leaders) show a significant, sustained decrease starting on January 8, 2021. Simultaneously, tweets from (**B**) a community of right-wing influencer accounts and (**C**) a community of right-wing commentator accounts became an average of 1.70 and 1.30 times more likely to be retweeted per follower, respectively.

As with Prominent US Skeptics, focusing on the US Right Leaders community in isolation seems to suggest that Twitter’s deplatforming action was successful. Specifically, Figure 4A shows that the deplatforming operation was associated with a sharp decrease in content – relative to counterfactuals based on prior data – from a community that contained the sitting U.S. President’s Twitter account, *OR = 0.14, 95% CI: 0.11 — 0.17, p*<*0.001* (see Table S7). This decrease continued throughout the “five strikes” period, *OR = 0.36, 95% CI: 0.33 — 0.38, p*<*0.001*(Figure 2B). This decrease was partially reversed when Twitter reinstated the President’s account in November 2022, *OR = 1.85, 95% CI: 1.32 — 2.58, p*<*0.001* (these findings replicated when comparing US Right Leaders to US Left Leaders; see Table S11).

#### Deplatforming was associated with increased content proportions and virality in related accounts

These outcomes were not replicated in other communities on the US political right. Specifically, Figures 3B and C show significant increases, rather than the absence of changes or even decreases that would be expected had the deplatforming action been fully successful, in content proportions from other right-wing communities, representing right-wing influencers, *OR = 3.58, 95% CI: 3.34 — 3.83, p*<*0.001*, and right-wing commentators, *OR = 2.74, 95% CI: 2.64 — 2.84, p*<*0.001*, during the “five strikes” period. Moreover, virality of tweets from the community containing the President’s account, *RR = 1.16, 95% CI: 1.13 — 1.20, p *< *0.001*, right-wing influencers, *RR = 1.12, 95% CI: 1.09 — 1.15, p *< *0.001*, and right-wing commentator accounts, *RR = 1.88, 95% CI: 1.84 — 1.92, p *<* 0.001* increased during the “five strikes” period see Figures 4B and C, and 2B (these results were not uniquely attributable to the decrease in followers following deplatforming; see Supplementary Information, Figure S3).

#### Deplatforming was consistently associated with information quality decreases and potential reactance

We found that information quality decreased in all three right wing communities by the end of the “five strikes” period relative to SARIMAX-generated counterfactual trends (see Figure 2C), $\Delta _{US Right Leaders}$ =*−0.04, 95% CI: −0.05 — −0.03, p *<* 0.001; *$\Delta _{US Right Influencers}$ =*−0.04, 95% CI: −0.05 — −0.03, p *< *0.001*; $\Delta _{US Right Commentators}$ =*−0.07, 95% CI: −0.08 — −0.06, p *< *0.001* and corresponding left-wing comparator groups, $\Delta _{US Right Leaders}$ =*−0.03, 95% CI: −0.04 — −0.02, p* < *0.001*; $\Delta _{US Right Influencers}$ =*−0.04, 95% CI: −0.05 — −0.03*, p < *0.001*; $\Delta _{US Right Commentators}$ =*−0.06, 95% CI: −0.07 — −0.05, p *< *0.001* (see Table S11). Furthermore, within the US Right Leaders community, the proportion of content containing political commentary, such as concerns about censorship, increased significantly by 21% relative to SARIMAX-generated counterfactuals and by 69% relative to US Left Leaders, suggesting that this increase was likely not due solely to an increase in standard political speech associated with events such as elections ($p<0.001$ in both cases; see Table S11).

## Discussion

During the COVID-19 pandemic and the 2020 US elections, Twitter — a social media platform that was widely used by journalists, government officials, and other public opinion leaders^52^ — implemented several policies intended to reduce misinformation spread. However, our results show that content violating Twitter’s medical misinformation policies did not decrease as intended, and may have additionally triggered a backlash. At best, Twitter’s policies were efficacious in the short term and ineffective — and possibly even counterproductive — in the long-term. Specifically, we observed that content proportions and virality increased, rather than decreased, in all vaccine skeptical communities during the “five strikes” phase – a period lasting almost two years. Additionally, URL domain quality decreased relative to trends derived from SARIMAX models fit to prior data and comparators during the “five strikes” phase for all vaccine skeptical communities, meaning that the content produced by these accounts appears to have become more misinformative. Furthermore, discussion of content expressing vaccine skepticism does not appear to have decreased in vaccine skeptical communities and actually increased in pro-vaccine communities, suggesting that — despite Twitter’s policies — vaccine skeptics, rather than vaccine promoters, successfully set the agenda for discussion topics on Twitter. Finally, Twitter’s policies may have triggered a backlash, as indicated by increases in the proportion of political and civil liberties topics among vaccine skeptical accounts.

Our findings move beyond prior work in several important ways. Whereas prior work appears to suggest that deplatforming operations might have been successful in the short-term^36^, our findings make it impossible for us to conclude that Twitter’s policies were efficacious in the long term. Furthermore, our findings move beyond prior work demonstrating that Facebook’s attempts to control vaccine misinformation during the COVID pandemic were not efficacious^31^ by enabling us to disentangle the effects of content removal from those of deplatforming. Some have speculated that deplatforming is especially efficacious — especially on Twitter — whereas content removal may be less so^36^. Our results suggest that content removal does indeed appear to have limited efficacy; however, contrary to prior work, we find that deplatforming appears to be counterproductive, both when carried out incrementally and suddenly. Finally, our analysis also moves beyond prior work by examining the effects of deplatforming on multiple communities on Twitter. We find that deplatforming appears to be associated with increases in content and virality, and decreases in URL quality in communities that are connected to, yet distinct from, the accounts that are targeted for removal. Although it is now well-documented that online communities, when deplatformed, may reconstitute on other platforms that are outside of the original platform’s control^30,31,37,38,53–60^ deplatforming is often considered successful because fringe platforms have smaller audiences, substantially reducing the potential for exposure to harmful content among most users. However, we show that content and account removal appear to have been associated with increases in vaccine skeptical content and engagement, and decreases in information quality *on the same, mainstream, platform*.

These results highlight the critical need to explain the conditions under which content moderation will be effective. Here, we observe that social media platforms’ architectures^39^ — which determine how information is allowed to flow between entities such as social media accounts — provides unique explanatory power. Specifically, Twitter’s architecture — an ahierarchical network — is among the most flexible, but also least controllable, of social media platform structures^61^. Indeed, Twitter’s design goal — as reflected in its 2012 mission statement — is “[t]o give everyone the power to create and share ideas and information instantly, without barriers.”^62^. Accordingly, we expect policies designed to impose barriers on information flow to have limited success.

Our theory suggests that Twitter’s architecture makes it especially difficult to moderate content effectively. If so, the attempts to moderate content on novel platforms possessing similar architectures, such as Bluesky and Mastodon, may face similar difficulties. Our findings suggest that Twitter is even more difficult to moderate than Facebook. The latter platform possesses a layered hierarchy^31^, which our theory associates with only moderate flexibility, and indeed, attempts to moderate content on Facebook have had mixed results, with a single internal study claiming that a massive deplatforming operation reduced the spread of hateful content to specific audiences^32^, but other studies finding no effect of content moderation efforts, including content removal, downranking content, and either temporarily or permanently suspending accounts that engaged in multiple violations on either audience engagement, content volumes, or both^31,33,34^. In contrast, prior research seems to suggest that content moderation efforts may be more effective on platforms such as Reddit^26,27,29,63–65^, whose architecture more closely resembles a tree-structured hierarchy^66^, and which our theory associates with less flexibility^39^.

Limitations to this study include that only tweets pertinent to COVID vaccines were studied. We can therefore only make claims about the efficacy of Twitter’s actions as they pertain to COVID vaccine misinformation, although Twitter’s medical misinformation policies were primarily designed to target these topics. Actions taken by Twitter to address misinformation about other topics that do not pertain to COVID vaccines are therefore beyond the scope of this work. We do not make claims about behaviors in private spaces, such as direct messages; however, this mechanism of information transmission is inconsistent with the primary use of Twitter, whose leadership conceives of it as a “digital town square”^67^. We also cannot rule out the possibility that vaccine promoting communities contained some vaccine skeptical content, and vice versa; however, the existence of parallel vaccine discourses on Twitter has long been documented^51^, suggesting that vaccine promoting communities primarily promoted vaccination and vaccine skeptical communities primarily promoted vaccine skepticism. Additionally, it is possible that some topics that were labeled as expressing “vaccine skepticism” actually expressed political commentary or vice versa. However, we found that such misclassifications were both infrequent and symmetric (i.e., disagreement on topic labels expressing skepticism was about as likely as disagreement on topic labels expressing commentary). Given that we did not observe changes in proportions of vaccine skeptical topic proportions, but we did observe increases in commentary topics — our results appear to be robust to any ambiguity resulting from misclassification. In fact, the bidirectional noise likely introduces conservative bias into our conclusions because true increases in skepticism might be partially masked by misclassifications into commentary. This interpretation strengthens our confidence that we are not overstating the prevalence or growth of vaccine-skeptical content. On the other hand, we regard the presence of potential reactance as tentative. Furthermore, we do not distinguish between posts made by individual users, institutions, and social bots. However, Twitter’s policies were not restricted to any one type of account; thus, our attempts to evaluate their efficacy must consider their effects on content shared on the platform as a whole.

A final limitation arises from the theoretical possibility that our models cannot account for unobserved, group-specific events that occurred at the same time as Twitter’s interventions if they were not reflected in the data on interventions. For example, if a vaccine skeptical community responded to a fringe news event that did not resonate with its pro-vaccine comparator, such asymmetric reactions might, in theory, confound estimates of policy impact. Although this is a limitation in theory, we find it to be highly implausible in practice for several reasons. First, this hypothetical event would need to be perfectly timed to align with Twitter’s policy interventions. Second, it would have to be strongly asymmetric, affecting only vaccine-skeptical communities. Third, the effect of this event would have to be large enough to more than cancel any policy-induced decrease. Fourth, it would have to have caused a sustained shift in the behavior of those communities (i.e., it would introduce non-stationarity into the time series for those communities). In effect, if we assume that the policy was successful, its effects would have to have been masked by a precisely-timed simultaneous increase in vaccine skeptical content that was larger in magnitude while having no observable effect on pro-vaccine comparators. At best, the effect of such a policy was too small to detect. Furthermore, given that Twitter’s policies were implemented over the span of over two years, the effects of any hypothetical masking event would have had to have persisted for the same period of time. However, we tested for, and found no evidence of, non-stationarity, meaning that the effects of such a hypothetical event would not have persisted over time and could not have caused the observed long-run behaviors. Finally, in our LSDV analysis, we interacted community identifiers with intervention variables that explicitly capture the intensity of Twitter’s policy enforcement, allowing for differential responses across groups. Taken together, an alternative explanation positing that Twitter’s actions caused decreases in medical misinformation seems implausible in light of the observed increases in vaccine skeptical content, virality, low-quality weblinks, and political/moral commentary. Nevertheless, we interpret our findings conservatively, emphasizing that our results reflect associations consistent with an ineffective policy rather than positing that the policy caused the observed increases.

Our results provide crucial context to recent reductions in content moderation efforts by major social media platforms^68^. Even in cases where moderation was well-resourced and sustained over time, as with Twitter’s COVID-19 vaccine misinformation policies, the outcomes were mixed at best and at times counterproductive. Our findings therefore provide a critical evidence base for platforms and policymakers to consider as they grapple with whether and how to re-engage with problematic online content. Specifically, we may expect the success of these efforts to vary across platforms in a manner that is mediated by system architecture.

Overall, our results point towards the need for development of scientific theory underlying how a platform’s designed architecture can facilitate or inhibit efforts to ensure that its users are able to access information that they need to make informed decisions. Such a theory, when fully developed, can help users to select social media platforms that better align with their goals and values, while facilitating users’ access to information required to make scientifically-informed decisions.

## Methods

In accordance with Federal regulations for the protection of human research subjects, 45CFR46.101(b), this research qualifies as exempt under category No. 4 as determined by the Johns Hopkins Homewood Institutional Review Board (IRB; No. 2011123) and the George Washington University IRB (No. 180804).

### Data

Our dataset consists of over 400 million tweets from over 6.7 million Twitter accounts collected in real time between February 6, 2020 – shortly after the first case of COVID-19 was detected in the USA – and December 15, 2022, shortly after Twitter ceased enforcing its misinformation removal policies^69^. These tweets were collected using the Twitter public keyword streaming API to download all tweets containing the following COVID-19 related keywords: coronavirus, wuhan, 2019ncov, sars, mers, 2019-ncov, wuflu, COVID-19, COVID19, COVID, covid-19, covid19, covid, SARS2, and SARSCOV19. We matched (case-insensitive) every downloaded tweet against the above keywords, including if they appear as hashtags. We downloaded all tweets in this dataset between February 6, 2020, December 17, 2022 and retained those that contained the string “vacc” or “vax”.

We study three primary outcome variables:

1. daily and monthly relative activity of each Twitter community
2. daily and monthly virality, measured as retweets per follower, of each Twitter community
3. the daily and monthly quality of URLs shared in each community.

A secondary analysis examined the proportion of tweets in each community that discussed topics expressing vaccine skepticism, topics promoting vaccination, and topics that framed vaccination in terms of civil liberties or other political or moral issues. Our dataset includes all tweets by accounts in each community. The strength of this approach is that it provides a comprehensive analysis of activity on the platform regardless of sources. The limitation is that non-human accounts, such as organizations or bots, are included in our dataset; however, Twitter’s policies also targeted these non-human accounts. Therefore, it is reasonable to include them.

### Procedure for identifying Twitter communities

We used our full dataset to construct a weighted retweet network. Each unique Twitter account in our dataset was represented by a node in this network and pairs of nodes were connected by a weighted arc if one account retweeted a message from another account at least once, with arc weights corresponding to the total number of retweets. We next performed a 3-core decomposition of the resulting network, removing all nodes that were not connected to at least 3 others. Finally, we used the Louvain method for community detection^70^ to extract 25 communities. For each community, we identified the 25 most active users and used these to assign that community a label based on information shared in each account’s “description” field (see Table S12).

### Missing data

Data were occasionally missing due to server issues^69^. We identified days on which data were missing by determining which days had no recorded user-authored tweets. We dropped dependent measures for these days. To account for the fact that the corresponding server outages may have started during the day before or lasted until the day after – leading to partial counts – we also dropped dependent measures from the days immediately before or immediately after these identified outages. For each dependent measure, we conducted linear interpolations to impute these missing data. The dates for which data were dropped are: March 4-6, 13-17, and 20-24, 2020; September 26-29, 2020; February 6-8, and 20-22, 2021; March 6-8 and 20-22, 2021.

### Design

The objective of this study was to answer the following three research questions:

1. Were Twitter’s content removal and “five strikes” policies associated with a proportional decrease in public vaccine skeptical content?
2. Did virality of remaining content decrease after these policies were implemented?
3. Did misinformation decrease when these policies were implemented?

A major challenge to our analysis is that Twitter’s policies coincided with several relevant world events including the global rollout of COVID vaccines, the emergence of new COVID variants, and significant political events, such as a large riot at the US Capitol on January 6, 2021. These events could potentially confound analyses, precluding causal comparisons. We therefore conducted two separate quasi-experimental analyses designed to facilitate causal claims subject to specific assumptions.

#### Comparative interrupted time series design

**Control Group Selection**

Our first quasi-experimental design relies on non-equivalent control groups. Specifically, we used a Comparative Interrupted Time Series Design (CITS) design with a non-equivalent control group – one of the strongest quasi-experimental designs available^71^. This design enabled us to estimate the effects of changes to Twitter’s policies. Specifically, any changes to observed data affecting anti-vaccine content that are not due to Twitter’s policies – e.g., external news about vaccine trials – are expected to affect both pro- and anti-vaccine communities equally since they are both focused on vaccination.

**Research Question 1: Did the Proportion of Vaccine Skeptical Content Decrease?**

To answer research question 1, we measured the total number of daily tweets (including retweets and quote tweets) produced by accounts in each community. We divided this number by the total number of daily tweets produced by all 25 communities, yielding a proportion between 0 and 1. We next applied a logistic transform to this number to control for floor and ceiling effects. This measure provides a daily snapshot of the activity of each community relative to all other communities.

**Research Question 2: Did Virality of Vaccine Skeptical Tweets Decrease?**

To answer research question 2, we first calculated the total number of retweets per follower for each user-authored tweet in our dataset. Retweet counts were current as of December 17, 2020 (the last day of our data collection), and follower counts were calculated from the time of each tweet’s posting. We used Laplace smoothing with $\alpha = 1$ to account for user-authored tweets that had not been retweeted or accounts with no followers. Since the resulting distribution of both retweets and followers was highly skewed, we next applied a log transform to the resulting proportions. Finally, we averaged this statistic across all user-authored tweets in each community per day.

**Research Question 3: Did Vaccine Skeptical Content Become Less Misinformative?** To answer research question 3, we used two convergent approaches. First, we used regular expressions to extract all URLs contained in each tweet (including retweets and quote tweets), lengthening shortened URLs if necessary. For each URL we used the TLDExtract python package^72^to extract the top-level domain (TLD) and suffix for each URL (for example, the top-level domain of www.example.com/this-is-an-example.html is example.com). We next assigned these domains a quality rating between 0 and 1 using the list compiled by Lin and colleagues^73^. We use this list because it has significant coverage, combines ratings from multiple sources, has demonstrated high interrater reliability across these sources, and is among the most comprehensive and reliable measures of domain quality. We retained the domain quality score with the lowest rating for tweets containing multiple URLs. Finally, for each community, we calculated daily domain quality ratings by averaging across all rated tweets in that community.

In our secondary analysis, we examined the content of tweets by conducting a comprehensive topic classification of tweets in our dataset. Specifically, we fit 25 BERTopic models^74^ to all unique tweets (i.e., excluding retweets and quote tweets) from each community. We then merged these 25 models into a single model using BERTopic’s native “merge models” function, resulting in 19,760 distinct topics. Finally, we assigned each tweet to its modal topic. Retweets were assigned to the topic of their original tweet, and we used the merged BERTopic model to perform post-hoc inference on all quote tweets.

Once all tweets were assigned to a topic, we used the OpenAI ChatGPT-4o model via the ChatGPT API to classify each topic into categories from the Jamison et al. vaccine discourse typology^75^ (see Supplementary Information for detailed prompt instructions). To assess classification reliability, we queried the model five times per topic and used Krippendorff’s $\alpha$ to measure agreement across these independent ratings. The model demonstrated high reliability ($\alpha$= 0.87), indicating strong consistency in topic labeling.

Based on the aggregated classifications, we grouped tweets into the following categories:

- Vaccine skepticism – Content for which all five ChatGPT instances assigned the associated topic into one of the following categories:
  - Alternative medicine
  - Conspiracy
  - Safety concerns
- Vaccine promotion – Content for which all five ChatGPT instances assigned the associated topic into one of the following categories:
  - Pro-science
  - Provaccine policy
  - Criticizing antivaccine beliefs
  - Promotion
  - Safety and efficacy
- Commentary – Content for which at least one ChatGPT instance assigned the associated topic into at least one of the following categories, and none of the remaining instances assigned the topic to a “promotion” or “skepticism” category:
  - Civil liberties
  - Morality
  - Politics
- Other – Tweets not fitting into the above categories or with disagreement between ChatGPT instances.

We then calculated the proportion of tweets in each category by community over time after applying Laplacian smoothing. As with content proportions, we applied a logistic transform to this number to control for floor and ceiling effects.

As a robustness check, two of the study’s authors (DAB and AMJ), annotated a simple random sample of 300 topics. Agreement between ChatGPT ratings and human annotators indicated sufficiently high agreement to support the use of these annotations for analytic conclusions, $\alpha$ *= 0.71* and was comparable to agreement between human annotators without including ChatGPT ratings, $\alpha$ = *0.75*. We also examined where disagreements between ChatGPT and human raters were most likely to occur, and found that the most frequent confusion — both between human annotators and between humans and ChatGPT — involved the boundary between skepticism and commentary. These categories, while theoretically distinct, often overlap in practice, such as when topics use vaccine safety or efficacy concerns to justify taking political positions. Importantly, we found that this confusion was roughly symmetric – human raters and ChatGPT did not favor skepticism labels over commentary labels or vice versa (see Supplementary Information for a full description of our annotation procedures).

**Statistical Analysis**

To answer our research questions, we extracted a total of 12 daily time series for each dependent measure. Six of these time series reflect activity in six different vaccine skeptical communities. Each of these six time series was compared to a time series from a thematically-similar pro-vaccine community. For example, a community opposing vaccination in Canada was compared to a community promoting vaccination in Canada (see Table S12 for a list of communities and their comparators). In order to minimize the possibility that our findings are spurious due to the choice of comparators, we estimated a separate model with fixed effects for comparator, using all 25 comparators, aggregated to the monthly level. Our results replicate.

The pre-policy period was defined as February 7, 2020, through December 20, 2020 – the first day of the workweek after Twitter stated its intention to remove vaccine misinformation under its medical misinformation policy [https://blog.x.com/en_us/topics/company/2020/covid19-vaccine]. The “content removal” phase was defined as the following day through February 28, 2021. The “five strikes” phase was defined as March 1, 2021 — when Twitter announced a new policy to reduce misinformation on the platform by imposing progressively harsher sanctions against, and ultimately removing, accounts that repeatedly posted misinformation [https://blog.x.com/en_us/topics/company/2021/updates-to-our-work-on-covid-19-vaccine-misinformation] — through November 22, 2022, the day before Twitter suspended enforcement of its medical misinformation policy^43^.

We conducted interrupted time series analyses using Seasonal Autoregressive Integrated Moving Average with eXternal regressors (SARIMAX) models fit to daily time series for each dependent variable^76^. This model is specified as:

1 $$\begin{aligned} \Theta (L)^p\Theta (L^S)^P\Delta ^d\Delta ^D_Sy_t=\Phi (L)^q\Phi (L^S)^Q\Delta ^d\Delta ^D_S\epsilon _t+\sum _{i=1}^{n} \beta _ix^i_t \end{aligned}$$

where $y_t$ is the quantity being predicted (e.g., the daily number of posts), $\epsilon _t$ is the error at time t, $\Theta (L)^p$ is a p-order polynomial function of L capturing autoregressive terms (i.e., $L^ny_t=y_{t-n}$) and $\Phi (L)^q$ us a q-order polynomial function of L capturing moving average terms (i.e., $L^n\epsilon _t=\epsilon _{t-n}$). In addition, $y_t^{[d]}|=\Delta ^dy_t=y_t^{[d-1]}-y_{t-1}^{[d-1]}$ where $y_t^[0]=y_t$ and d is the order of differencing. By analogy, $\Theta (L^S)^P$ is a P-order seasonal autoregressive term and $\Phi (L^S)^Q$ us a Q-order seasonal moving average term both with period S, and $\Delta ^D_S$ is a seasonal lag term of order S (i.e., $\Delta ^D_Sy_t=y_t^{[d-1]}-y_{t-S}^{[d-1]}$). Finally, $x_t^i$ is an external regressor, such as the daily proportion of tweets in a given community.

We fit all SARIMAX models to daily data up to, but not including, the first date of the policy being evaluated. To do so, We used the auto_arima function in the pmdarima python package^77^ using default settings. When fitting these models, we included fixed effects as binary dummy variables to account for known prior changes in Twitter’s misinformation enforcement activities as reported in blog posts and press releases [https://blog.x.com/en_us/topics/company/2020/covid-19] (see Table 1)

**Table 1:** Throughout the COVID-19 pandemic, Twitter announced several measures that were designed to assist users to find high quality health information on their blog. We included dummy variables for each of these announcements in order to isolate the effects of Twitter’s vaccine misinformation and “five strikes” policies from these other measures.

Fixed Effect	Start	End
Initial period	Friday, February 7, 2020	Tuesday, March 3, 2020
Global COVID-19 search prompt & event page	Wednesday, March 4, 2020	Sunday, March 15, 2020
Updated policies & automated tech	Monday, March 16, 2020	Thursday, March 26, 2020
Broadened definition of harm	Friday, March 27, 2020	Wednesday, April 1, 2020
Updated ad policy	Thursday, April 2, 2020	Tuesday, April 21, 2020
Unverified claims violate policies	Wednesday, April 22, 2020	Sunday, May 10, 2020
New labels and warning messages	Monday, May 11, 2020	Sunday, May 17, 2020
COVID-19 tab in explore	Monday, May 18, 2020	Monday, July 13, 2020
Clarifying assessment of misleading information	Tuesday, July 14, 2020	Saturday, December 19, 2020
Vaccine misinformation removal policy	Sunday, December 20, 2020	Thursday, January 7, 2021
Mass deplatforming	Friday, January 8, 2021	Sunday, February 28, 2021
“Five strikes” policy	Monday, March 1, 2021	Tuesday, November 22, 2022
Former president’s account reinstated	Friday, November 18, 2022	Saturday, December 17, 2022
Medical misinformation policy suspended	Wednesday, November 23, 2022	Saturday, December 17, 2022

According to the CITS approach’s causal interpretation, Twitter’s content and account removal policies in anti-vaccine communities were effective if they caused greater deviations from counterfactual trends that were derived from these communities’ prior data than from those derived from pro-vaccine communities. We therefore used these models to generate counterfactual forecasts for each dependent variable assuming no policy changes. We calculated the percent difference between these counterfactual projections and observed post and engagement counts. We consider a policy to have been effective if it was consistently associated with a reduction in content beyond the 95% confidence bounds of these projections.

Pro-vaccine comparators make ideal non-equivalent control groups because, like anti-vaccine communities, they contain users who are motivated to post about vaccines and would therefore respond to exogenous factors, such as the news cycle, in the same way; however, platforms’ policies were not designed to target pro-vaccine content. A statistically significant difference in anti-vaccine, but not pro-vaccine, communities, or between anti- and pro-vaccine communities effect sizes – indicates that it is more likely to have been Twitter’s policies, and not some contemporaneous event, which caused the observed change.

Having fit SARIMAX models, we used the procedure recommended by Fanshawe et al.^78^ to compare observed data to counterfactual trends by calculating 1,000 forecasted time series using the “simulate” function in the statsmodels python package^79^. For each forecast, we calculated $M_k=\sum _{t=n+1}^{n+k}\frac{\hat{y_t}}{k}$, where n is the total number of days before a given policy was implemented and k is the total number of days forecasted. Thus, $M_k$ represents the average of the forecasted values over the post-policy period. Given 1,000 such forecasts, we were able to calculate the mean, $\hat{m}_k$, and standard deviation, $\hat{s}_k$, of $M_k$. Given a vector of post-policy data, $y_t$, we calculated a Z statistic for each post-policy time series as $Z=\frac{\sum _{t=n+1}^{n+k}\frac{y_t}{k}-\hat{m}_k}{\hat{s}_k}$, where the numerator of this quantity reflects the average difference between actual and simulated post-policy means and the denominator reflects the standard deviation of this difference. Consequently, the 95% confidence intervals for this quantity can be estimated as

2 $$\begin{aligned} CI=\sum _{t=n+1}^{n+k}\frac{y_t}{k}-\hat{m}_k\pm \mathcal {N}(1+\frac{\alpha }{2})*\hat{s}_k \end{aligned}$$

where $\mathcal {N}(1+\frac{\alpha }{2})$ is the inverse cumulative distribution function for a Type I error rate of $\alpha$. For this study, $\alpha$=0.05, and $\mathcal {N}(1+\frac{\alpha }{2})=1.96$ unless otherwise specified (e.g., when correcting for multiple comparisons).

We calculated the effect of Twitter’s policies on vaccine skeptical communities relative to pro-vaccine communities as

3 $$\begin{aligned} Z=\frac{(\sum _{t=n+1}^{n+k}\frac{y_{t,anti}}{k}-\hat{m}_{k,anti})-(\sum _{t=n+1}^{n+k}\frac{y_{t,pro}}{k}-\hat{m}_{k,pro})}{\sqrt{(\hat{s}_{k,anti})^2-(\hat{s}_{k,pro})^2}} \end{aligned}$$

with the numerator of this quantity reflecting the average relative difference and the denominator reflecting the standard deviation of this difference. We conducted analyses of tweet proportions using logit-transformations to account for multinomial variance-covariance structures for which exponentiating average daily differences yields odds ratios. Additionally, analyses of virality data (retweets per follower) were conducted using log-transformations; thus, exponentiating these average differences yields a relative risk. Analyses conducted on domain quality data were not transformed since domain quality ratings were generated using principal component analysis^73^, which assumes a linear additive model.

A statistically significant difference between vaccine skeptical and pro-vaccine effect sizes indicates that it is more likely to have been Twitter’s policies, and not some contemporaneous event, which caused the observed change. In contrast, statistically significant differences in both vaccine skeptical and pro-vaccine communities may indicate either the effects of some exogenous event or, alternatively, that Twitter’s policies affected both types of content. By using this approach, we were able to make inferences about the effects of Twitter’s policies that would not be possible using vaccine skeptical data alone.

Importantly, our models do not require making a “parallel path” assumption – i.e., that different communities would have evolved in the same way except for implementation of Twitter’s policy – a necessary prerequisite of the widely-used Difference in Differences (DiD) design. Unlike the DiD approach, our approach also does not require us to assume a that the relationship between Twitter’s policies and activity in pro- and anti-vaccine accounts over time, or to assume a “burn-in” period of arbitrary length during which Twitter’s policies were being rolled out. Rather, this approach allows us to assess the net effect of Twitter’s policies throughout each phase. Furthermore, this model does not require us to assume that Twitter implemented their policies consistently throughout the post-policy period, but may have instead engaged in varying levels of enforcement.

Our primary hypothesis is that Twitter’s interventions were ineffective. However, absence of evidence is not evidence of absence. The strongest evidence against the policy’s efficacy would therefore be a relative *increase* in misinformation among vaccine skeptical communities at the time of the policy’s implementation, both relative to trends derived from prior data and relative to pro-vaccine comparators. Although we cannot clearly attribute such an increase to the policy itself, it would be clear evidence that the policies failed to reduce misinformation as intended.

Statistical analyses were performed using python’s pmdarima^77^ and statsmodels^79^ packages. All tests were 2-sided and a P value of 0.05 or less was considered statistically significant.

#### Fixed effects regression design

In addition to evaluating a discontinuous change in content removal policies, we conducted a second analysis, which serves three purposes.

1. To determine the relationship between *intensity* of policy enforcement and our dependent variables: Whereas the CITS design excels at disentangling the effects of a discontinuous change in policy at a specific point in time, it does not account for within-policy variation, as might have occurred during the “five strikes” period when Twitter progressively sanctioned misinformative accounts.
2. As a robustness check: Although the CITS design can determine whether changes in vaccine skeptical communities were significantly different from their pro-vaccine comparators, the length of the evaluation period calls into question whether it was the change in policy, *per se*, that caused any observed changes in outcome variables, e.g., because Twitter may have progressively implemented its policy changes over time or because external events that might have incentivized greater policy enforcement, such as public critiques of Twitter made by prominent public figures.
3. To disentangle the effects of overlapping policy regimes: The CITS design is based on the identification of two discrete policy regimes – a content removal phase, and the “five strikes” phase during which accounts were also subject to removal. However, Twitter continued to remove content during the “five strikes” phase, meaning that the CITS analysis cannot uniquely attribute changes observed during the “five strikes” phase to account removal.

To address these limitations, we used publicly-available monthly data from July, 2020, through September, 2022, documenting the number of tweets and accounts that had been removed for violating Twitter’s medical misinformation policy [https://transparency.x.com/en/reports/covid19.html]. We used these data as predictors in a Least Squares Dummy Variable (LSDV) approach, with a dichotomous dummy for each Twitter community. Specifically, we recalculated the three dependent variables used in the CITS analysis when aggregated to the monthly level, and used Twitter’s “Accounts suspended” and “Content removed” numbers reported by Twitter to predict these dependent variables. In order to account for potential endogeneity of our predictors (e.g., that Twitter’s enforcement intensity might have increased as a result of increases in misinformation), we lagged all predictor variables by one month such that transparency data at time t-1 was used to predict dependent variables at time t. As such, dependent variables were dated August, 2020, through October, 2022. We also included a first-order lagged dependent variable to capture potential autoregression. Finally, in order to examine differences in how our predictors affected different communities, we examined statistical two-way interactions between each predictor and each dummy variable. We used augmented Dickey-Fuller tests to test for stationarity of residuals, and found that residuals were stationary. All standard errors were estimated using heteroskedasticity- and autocorrelation-consistent (HAC) estimators to account for any potential remaining autocorrelation and heteroskedasticity of residuals. Statistical analyses were performed using python’s statsmodels^79^ package. All tests were 2-sided and a P value of 0.05 or less was considered statistically significant.

## Supplementary Information

Supplementary Information.

## Data Availability

Tweet IDs for the data used in this paper are available at https://doi.org/10.5281/zenodo.3735015
