# Supplementary material for: Explaining Twitter’s inability to effectively moderate content during the COVID-19 pandemic
Source: Sci Rep. 2025 Oct 15;15:36096. doi: 10.1038/s41598-025-20033-6 (PMC12528495; doi:10.1038/s41598-025-20033-6)
Supplement: Supplementary file 1 — Supplementary Information. [file 41598_2025_20033_MOESM1_ESM.pdf]

Supplementary Information for: Explaining Twitter's inability to effectively moderate content during the COVID-19 pandemic

Supplementary Figures

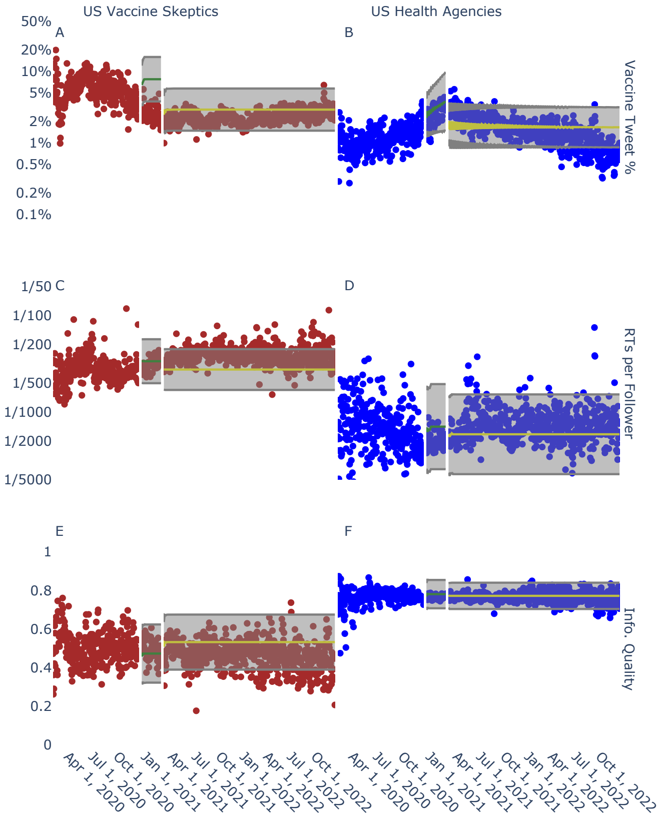

Figure S1: Time series plots showing activity generated by a cluster of accounts containing, and amplifying content from, US Vaccine Skeptics (dark red), compared to a cluster of accounts containing, and amplifying content from, US Vaccine Promoters (US Health Agencies). Each plot is segmented into three periods capturing the pre-policy period (before December 20, 2020), the content removal phase (December 20, 2020 through February 28, 2021) and the "five strikes" phase (March 1, 2021 through November 22, 2022). Green lines and yellow lines show pre-policy SARIMAX projections for the content removal and "five strikes" phases, respectively. 95% confidence intervals are shown. A) Daily proportions of tweets generated by a cluster of accounts amplifying content from US Vaccine Skeptics; B) daily proportion of tweets generated by a cluster of accounts amplifying content from US Health Agencies. C) daily average retweets per follower for tweets generated by communities retweeting US Vaccine Skeptics and D) US Health Agencies. E) daily average URL domain quality rating for tweets generated by communities retweeting US Vaccine Skeptics and F) US Health Agencies.

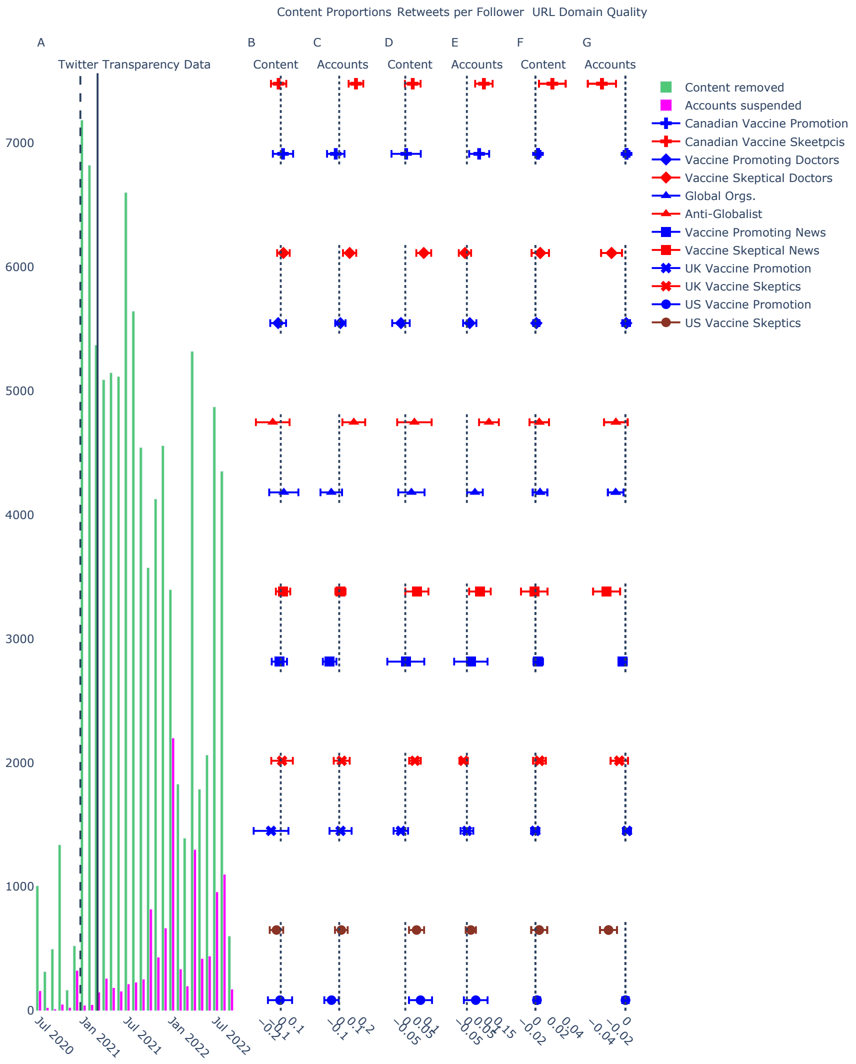

Figure S2: A) Twitter transparency data records the monthly number of posts and accounts removed for violating Twitter's medical misinformation policies. Regression coefficient estimates when predicting logit changes in monthly content proportions per cluster per B) piece of content removed C) account removed; log-transformed retweets per follower per D) piece of content removed E) account removed; Average monthly URL domain quality score per F) piece of content removed; G) account removed. All count data were aggregated to the level of the month and logarithmic transforms were applied to correct for data skew.

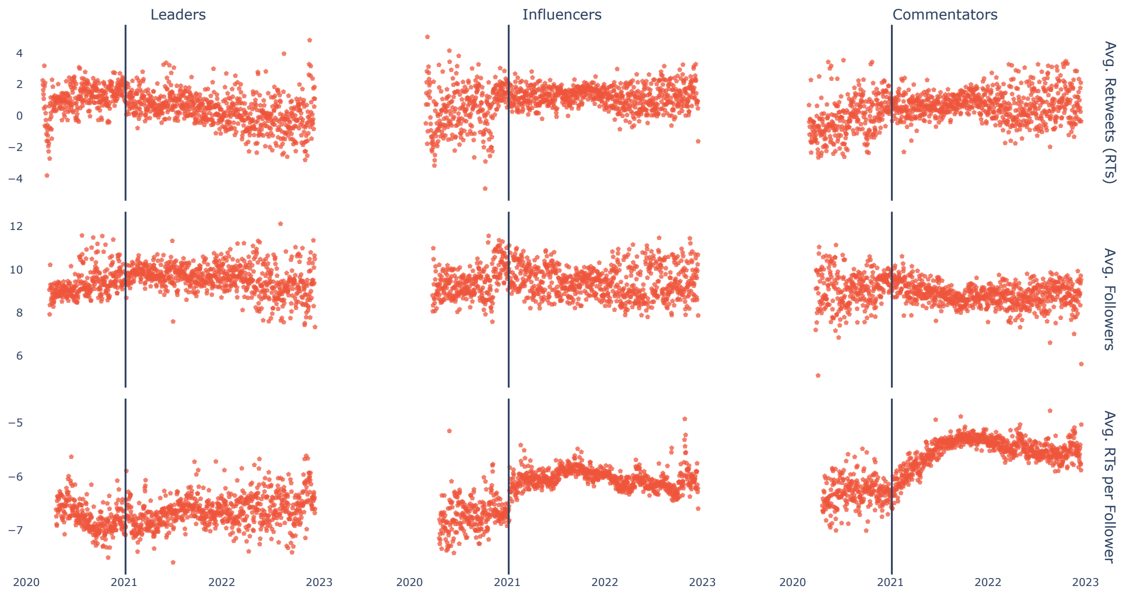

Figure S3: Increases in virality (Retweets per Follower, bottom row), were not uniquely attributable to a sudden increase in the number of retweets, or a sudden drop in the number of followers following Twitter's mass deplatforming on January 8, 2021 (sold black line)

Supplementary Tables

Table S1: Communicability Values for Tree-Structured Hierarchies, evaluated at n=3,686,697 – the total number of accounts in our data after applying 3-core network decomposition to identify communities.

Regular Trees; $O(n)$		
	One Layer	
Branching Factor	Equation	$log_{10}C^{TN}$
2	$C^{TN} = 9.88n - 23.39$	7.56
3	$C^{TN} = 13.02n - 51.73$	7.68
4	$C^{TN} = 16.92n - 95.45$	7.80
5	$C^{TN} = 21.63n - 152.93$	7.90
6	$C^{TN} = 27.63n - 285.37$	8.01
7	$C^{TN} = 33.45n - 295.78$	8.09
8	$C^{TN} = 41.85n - 474.30$	8.19
9	$C^{TN} = 51.97n - 742.20$	8.28
	Two Layers	
2	$C^{TN} = 26.85n - 127.17$	8.00
3	$C^{TN} = 35.37n - 266.52$	8.12
4	$C^{TN} = 46.00n - 518.94$	8.23
5	$C^{TN}C = 58.79n - 831.40$	8.34
6	$C^{TN} = 72.07n - 975.90$	8.42
7	$C^{TN} = 90.93n - 1608.03$	8.53
8	$C^{TN} = 113.76n - 2578.58$	8.62
9	$C^{TN} = 141.27n - 4035.02$	8.72
	Three Layers	
2	$C^{TN} = 39.53n - 280.89$	8.16
3	$C^{TN} = 52.07n - 588.66$	8.28
4	$C^{TN} = 67.08n - 954.74$	8.39
5	$C^{TN} = 86.57n - 1836.33$	8.50
6	$C^{TN} = 106.12n - 2155.46$	8.59
7	$C^{TN} = 133.89n - 3551.66$	8.69
8	$C^{TN} = 167.51n - 5695.32$	8.79
9	$C^{TN} = 208.82n - 8912.15$	8.88

Table S2: Communicability Values for Regular Lattice Structures, evaluated at n=3,686,697 – the total number of accounts in our data after applying 3-core network decomposition to identify communities.

	Lattices; $O(n)$	
Dimensionality	Equation	$log_{10}C^{TN}$
	One Layer	
2	$C^{TN} = (7.39\sqrt[d]{n} - 10.26)^d$	8.30
3	$C^{TN} = (7.34\sqrt[d]{n} - 9.91)^d$	9.15
4	$C^{TN} = (7.16\sqrt[d]{n} - 9.21)^d$	9.94
5	$C^{TN} = (6.89\sqrt[d]{n} - 8.46)^d$	10.63
	Two Layers	
2	$C^{TN} = (17.35\sqrt[d]{n} - 2.76)^d$	9.04
3	$C^{TN} = (14.09\sqrt[d]{n} - 8.40)^d$	10.01
4	$C^{TN} = (15.56\sqrt[d]{n} - 18.54)^d$	11.29
5	$C^{TN} = (16.92\sqrt[d]{n} - 24.03)^d$	12.55
	Three Layers	
2	$C^{TN} = (25.86\sqrt[d]{n} - 5.06)^d$	9.39
3	$C^{TN} = (18.70\sqrt[d]{n} - 12.48)^d$	10.38
4	$C^{TN} = (18.97\sqrt[d]{n} - 22.61)^d$	11.63
5	$C^{TN} = (27.50\sqrt[d]{n} - 46.07)^d$	13.58

Table S3: Communicability Values for Regular Torus Structures, evaluated at n=3,686,697 – the total number of accounts in our data after applying 3-core network decomposition to identify communities.

	Toruses; $O(n)$	
Dimensionality	Equation	$log_{10}C^{TN}$
	One Layer	
2	$C^{TN} = 54.60n - 9.39$	8.30
3	$C^{TN} = 403.78n - 502.27$	9.17
4	$C^{TN} = 2999.49n - 17994.72$	10.04
5	$C^{TN} = 22540.47n - 455929.19$	10.92
	Two Layers	
2	$C^{TN} = 148.46n - 73.81$	8.74
3	$C^{TN} = 1099.47n - 3761.86$	9.61
4	$C^{TN} = 8233.00n - 127165.56$	10.48
5	$C^{TN} = 61271.35n - 2478687.96$	11.35
	Three Layers	
2	$C^{TN} = 218.61n - 163.03$	8.91
3	$C^{TN} = 1618.94n - 8308.83$	9.78
4	$C^{TN} = 12122.82n - 280870.77$	10.65
5	$C^{TN} = 90220.08n - 5474681.77$	11.52

Table S4: Communicability Values for Regular Torus Structures, evaluated at n=3,686,697 – the total number of accounts in our data after applying 3-core network decomposition to identify communities.

	Toruses; $O(n)$	
Dimensionality	Equation	$log_{10}C^{TN}$
	One Layer	
2	$C^{TN} = 54.60n - 9.39$	8.30
3	$C^{TN} = 403.78n - 502.27$	9.17
4	$C^{TN} = 2999.49n - 17994.72$	10.04
5	$C^{TN} = 22540.47n - 455929.19$	10.92
	Two Layers	
2	$C^{TN} = 148.46n - 73.81$	8.74
3	$C^{TN} = 1099.47n - 3761.86$	9.61
4	$C^{TN} = 8233.00n - 127165.56$	10.48
5	$C^{TN} = 61271.35n - 2478687.96$	11.35
	Three Layers	
2	$C^{TN} = 218.61n - 163.03$	8.91
3	$C^{TN} = 1618.94n - 8308.83$	9.78
4	$C^{TN} = 12122.82n - 280870.77$	10.65
5	$C^{TN} = 90220.08n - 5474681.77$	11.52

Table S5: Communicability Values for Complete Structures, evaluated at n=3,686,697 – the total number of accounts in our data after applying 3-core network decomposition to identify communities.

	Teams; $O(e^n)$	
Number of Layers	Equation	$log_{10}C^{TN}$
1	$C^{TN} = 13.94e^n$	3,705,048.33
2	$C^{TN} = 163.67e^{\frac{n}{2}}$	1,849,776.64
3	$C^{TN} = 361.51e^{\frac{n}{3}}$	1,233,186.90

Table S6: Communicability Values for Scale-Free Networks with Average Degree = 1 and γ = 1.46 evaluated at n=3,686,697 – the total number of accounts in our data after applying 3-core network decomposition to identify communities.

Scale-Free Networks; $O(e^{\frac{n}{\gamma}})$		
Percentile	Equation	$log_{10}C^{TN}$
	One Layer	
2.5	$C^{TN} = 0.92e^{\frac{0.70n}{\gamma}}$	24,583.67
50.0	$C^{TN} = 62.94e^{\frac{0.77n}{\gamma}}$	26,289.57
97.5	$C^{TN} = 6518.29e^{\frac{0.85n}{\gamma}}$	28,008.56
	Two Layers	
2.5	$C^{TN} = 17.70e^{\frac{0.37n}{\gamma}}$	15,756.60
50.0	$C^{TN} = 139.47e^{\frac{0.41n}{\gamma}}$	16,889.05
97.5	$C^{TN} = 2020.98e^{\frac{0.45n}{\gamma}}$	18,183.69
	Three Layers	
2.5	$C^{TN} = 37.94e^{\frac{0.25n}{\gamma}}$	12,241.88
50.0	$C^{TN} = 209.23e^{\frac{0.28n}{\gamma}}$	13,035.62
97.5	$C^{TN} = 1143.98e^{\frac{0.31n}{\gamma}}$	14,075.13

Table S7: CITS results comparing vaccine skeptical communities to vaccine promoting comparators. Results include content proportions, retweets per follower, and URL domain quality ratings during the Content Removal and Five Strikes policy regimes. OR = Odds Ratio. RR = Risk Ratio. Δ = difference

Policy		Content Removal 95% CI				Five Strikes 95% CI			
		Content Proportions							
		OR	Lo	Hi	p	OR	Lo	Hi	p
Canada	Vaccine Promotion	1.04	0.60	1.78	0.45	1.20	1.01	1.41	0.02
	Vaccine Skeptics	1.23	1.05	1.45	0.006	5.31	5.04	5.60	<0.001
	Difference	1.19	0.67	2.09	0.28	4.45	3.73	5.29	<0.001
Doctors	Vaccine Promotion	1.52	1.36	1.70	<0.001	1.14	1.11	1.18	<0.001
	Vaccine Skeptics	1.26	1.14	1.40	<0.001	4.93	4.78	5.08	<0.001
	Difference	0.83	0.72	0.97	0.008	4.32	4.14	4.51	<0.001
Global	Global Orgs.	0.83	0.71	0.97	0.009	0.69	0.58	0.81	<0.001
	Anti-Globalist	0.89	0.81	0.99	0.02	2.99	2.88	3.10	<0.001
	Difference	1.07	0.89	1.29	0.22	4.36	3.66	5.19	<0.001
United States	Vaccine Promotion	0.88	0.58	1.34	0.27	0.88	0.79	0.97	0.005
	Vaccine Skeptics	0.33	0.28	0.38	<0.001	0.78	0.75	0.82	<0.001
	Difference	0.37	0.24	0.58	<0.001	0.89	0.80	0.99	0.02
News	Vaccine Promotion	1.10	0.99	1.24	0.04	0.38	0.36	0.41	<0.001
	Vaccine Skeptics	0.74	0.67	0.83	<0.001	1.56	1.33	1.84	<0.001
	Difference	0.67	0.58	0.79	<0.001	4.06	3.40	4.84	<0.001
United Kingdom	Vaccine Promotion	0.77	0.57	1.06	0.05	0.76	0.51	1.14	0.09

	Vaccine Skeptics	1.19	0.96	1.49	0.05	2.41	2.25	2.59	<0.001
	Difference	1.54	1.05	2.26	0.01	3.16	2.11	4.73	<0.001
						Retweets per Follower			
		RR	Lo	Hi	p	RR	Lo	Hi	p
Canada	Vaccine Promotion	0.83	0.75	0.92	<0.001	1.19	1.15	1.24	<0.001
	Vaccine Skeptics	1.10	1.01	1.19	0.02	1.97	1.92	2.03	<0.001
	Difference	1.32	1.16	1.51	<0.001	1.65	1.58	1.73	<0.001
Doctors	Vaccine Promotion	0.59	0.52	0.66	<0.001	1.05	1.01	1.10	0.01
	Vaccine Skeptics	1.16	1.07	1.27	<0.001	1.44	1.40	1.47	<0.001
	Difference	1.99	1.71	2.31	<0.001	1.37	1.30	1.44	<0.001
Global	Global Orgs.	2.34	1.32	4.14	0.002	0.94	0.87	1.02	0.08
	Anti-Globalist	1.00	0.83	1.21	0.50	3.18	2.99	3.40	<0.001
	Difference	0.43	0.23	0.78	0.003	3.38	3.04	3.75	<0.001
United States	Vaccine Promotion	0.88	0.73	1.07	0.10	1.25	1.19	1.32	<0.001
	Vaccine Skeptics	0.93	0.87	0.99	0.01	1.40	1.37	1.43	<0.001
	Difference	1.05	0.86	1.28	0.32	1.12	1.06	1.18	<0.001
News	Vaccine Promotion	0.80	0.71	0.91	<0.001	1.11	1.07	1.16	<0.001
	Vaccine Skeptics	1.05	0.90	1.21	0.28	1.73	0.97	3.06	0.03
	Difference	1.30	1.07	1.58	0.004	1.56	0.88	2.76	0.07
United Kingdom	Vaccine Promotion	0.98	0.91	1.05	0.29	0.72	0.70	0.73	<0.001
	Vaccine Skeptics	0.94	0.87	1.02	0.07	1.16	1.13	1.19	<0.001
	Difference	0.96	0.86	1.07	0.25	1.62	1.56	1.67	<0.001
						Domain Quality			
		Δ	Lo	Hi	p	Δ	Lo	Hi	p
Canada	Vaccine Promotion	0.00	0.00	0.01	0.17	0.00	0.00	0.00	0.47
	Vaccine Skeptics	0.09	0.05	0.12	<0.001	-0.02	-0.04	-0.01	<0.001

	Difference	0.08	0.04	0.12	<0.001	-0.02	-0.04	-0.01	<0.001
Doctors	Vaccine promotion	0.01	0.00	0.02	0.06	0.00	0.00	0.01	0.004
	Vaccine Skeptics	0.04	0.02	0.06	<0.001	-0.03	-0.03	-0.02	<0.001
	Difference	0.03	0.01	0.06	0.001	-0.03	-0.04	-0.03	<0.001
Global	Global Orgs.	0.00	-0.01	0.02	0.19	-0.02	-0.03	-0.02	<0.001
	Anti-Globalist	0.02	-0.01	0.04	0.09	-0.04	-0.05	-0.03	<0.001
	Difference	0.01	-0.02	0.04	0.18	-0.02	-0.03	-0.01	<0.001
United States	Vaccine Promotion	-0.01	-0.02	0.00	0.002	-0.01	-0.01	0.00	0.03
	Vaccine Skeptics	0.03	0.00	0.06	0.03	-0.07	-0.08	-0.05	<0.001
	Difference	0.04	0.01	0.08	0.004	-0.06	-0.07	-0.04	<0.001
News	Vaccine Promotion	-0.01	-0.02	0.00	0.04	-0.02	-0.02	-0.01	<0.001
	Vaccine Skeptics	-0.05	-0.07	-0.02	<0.001	-0.05	-0.06	-0.04	<0.001
	Difference	-0.04	-0.06	-0.01	0.004	-0.04	-0.05	-0.02	<0.001
United Kingdom	Vaccine Promotion	-0.01	-0.03	0.00	0.01	0.01	0.01	0.02	<0.001
	Vaccine Skeptics	0.05	0.02	0.08	<0.001	-0.03	-0.04	-0.02	<0.001
	Difference	0.06	0.03	0.09	<0.001	-0.04	-0.05	-0.03	<0.001
					% Vaccine Skeptical Topics				
		OR	Lo	Hi	p	OR	Lo	Hi	p
Canada	Vaccine Promotion	0.62	0.52	0.73	<0.001	1.48	1.40	1.56	<0.001
	Vaccine Skeptics	0.68	0.57	0.82	<0.001	1.10	1.04	1.17	<0.001
	Difference	1.10	0.86	1.41	0.22	0.75	0.69	0.81	<0.001
Doctors	Vaccine Promotion	0.84	0.72	0.99	0.02	1.64	1.56	1.73	<0.001
	Vaccine Skeptics	1.15	1.03	1.29	0.005	1.03	0.99	1.07	0.09
	Difference	1.37	1.13	1.67	<0.001	0.62	0.58	0.67	<0.001
Global	Global Orgs.	0.75	0.68	0.84	<0.001	1.25	1.22	1.28	<0.001
	Anti-Globalist	1.18	0.92	1.53	0.1	1.00	0.94	1.05	0.43
	Difference	1.57	1.19	2.07	<0.001	0.80	0.75	0.85	<0.001

United States	Vaccine Promotion	0.55	0.47	0.64	<0.001	1.16	1.10	1.22	<0.001
	Vaccine Skeptics	1.49	1.35	1.63	<0.001	0.84	0.81	0.86	<0.001
	Difference	2.71	2.26	3.24	<0.001	0.72	0.68	0.77	<0.001
News	Vaccine Promotion	0.69	0.37	1.28	0.12	0.85	0.28	2.54	0.38
	Vaccine Skeptics	1.20	1.05	1.38	0.004	1.17	1.12	1.22	<0.001
	Difference	1.75	0.93	3.32	0.04	1.38	0.46	4.14	0.28
United Kingdom	Vaccine Promotion	1.34	1.12	1.60	<0.001	1.29	1.22	1.36	<0.001
	Vaccine Skeptics	0.93	0.83	1.04	0.11	1.01	0.98	1.05	0.24
	Difference	0.69	0.56	0.86	<0.001	0.79	0.74	0.84	<0.001
		% Topics Containing Commentary							
		OR	Lo	Hi	p	OR	Lo	Hi	p
Canada	Vaccine Promotion	1.21	0.95	1.55	0.06	0.97	0.90	1.04	0.17
	Vaccine Skeptics	1.23	0.91	1.67	0.09	1.97	1.92	2.03	<0.001
	Difference	1.02	0.69	1.50	0.46	1.65	1.58	1.73	<0.001
Doctors	Vaccine Promotion	1.03	0.82	1.31	0.39	1.05	1.01	1.10	<0.001
	Vaccine Skeptics	1.21	0.98	1.49	0.04	1.44	1.40	1.47	<0.001
	Difference	1.17	0.86	1.60	0.16	1.37	1.30	1.44	<0.001
Global	Global Orgs.	1.20	0.94	1.53	0.07	0.94	0.87	1.02	<0.001
	Anti-Globalist	0.48	0.31	0.73	<0.001	3.18	2.99	3.40	<0.001
	Difference	0.40	0.25	0.65	<0.001	3.38	3.04	3.75	<0.001
United States	Vaccine Promotion	1.12	0.88	1.42	0.18	1.25	1.19	1.32	<0.001
	Vaccine Skeptics	0.75	0.64	0.88	<0.001	1.40	1.37	1.43	<0.001
	Difference	0.67	0.50	0.89	0.003	1.12	1.06	1.18	<0.001
News	Vaccine Promotion	1.45	0.94	2.24	0.05	1.11	1.07	1.16	0.07
	Vaccine Skeptics	1.86	1.49	2.31	<0.001	1.73	0.97	3.06	<0.001
	Difference	1.28	0.79	2.09	0.16	1.56	0.88	2.76	0.006
United Kingdom	Vaccine Promotion	1.03	0.79	1.33	0.42	0.72	0.70	0.73	<0.001

	Vaccine Skeptics	1.20	0.98	1.48	0.04	1.16	1.13	1.19	<0.001
	Difference	1.17	0.85	1.63	0.17	1.62	1.56	1.67	<0.001
					% Vaccine Promotion Topics				
		OR	Lo	Hi	p	OR	Lo	Hi	p
Canada	Vaccine Promotion	1.64	1.32	2.02	<0.001	0.94	0.86	1.02	0.06
	Vaccine Skeptics	2.03	1.70	2.42	<0.001	0.65	0.62	0.69	<0.001
	Difference	1.24	0.94	1.63	0.07	0.70	0.63	0.77	<0.001
Doctors	Vaccine promotion	1.53	1.25	1.88	<0.001	0.71	0.66	0.78	<0.001
	Vaccine Skeptics	0.94	0.81	1.09	0.21	0.77	0.74	0.81	<0.001
	Difference	0.61	0.48	0.79	<0.001	1.09	0.99	1.19	0.04
Global	Global Orgs.	1.37	1.16	1.62	<0.001	1.06	1.01	1.11	0.008
	Anti-Globalist	0.97	0.84	1.11	0.33	0.78	0.75	0.82	<0.001
	Difference	0.71	0.57	0.88	<0.001	0.74	0.69	0.79	<0.001
United States	Vaccine Promotion	2.74	1.81	4.15	<0.001	1.35	0.90	2.02	0.07
	Vaccine Skeptics	0.99	0.86	1.14	0.46	0.88	0.85	0.92	<0.001
	Difference	0.36	0.23	0.56	<0.001	0.65	0.44	0.98	0.02
News	Vaccine Promotion	1.24	0.92	1.67	0.08	1.16	0.91	1.48	0.11
	Vaccine Skeptics	0.71	0.57	0.88	<0.001	0.63	0.57	0.70	<0.001
	Difference	0.57	0.39	0.82	0.001	0.54	0.42	0.71	<0.001
United Kingdom	Vaccine Promotion	1.42	1.10	1.85	0.004	0.91	0.83	1.00	0.03
	Vaccine Skeptics	1.02	0.86	1.21	0.43	0.70	0.67	0.74	<0.001
	Difference	0.71	0.52	0.98	0.02	0.77	0.69	0.86	<0.001

Note. OR = Odds Ratio. RR = Relative Risk. Δ = difference. 95% CI = 95% Confidence Interval

Table S8: LSDV regression table predicting logit-transformed content proportions

	B	SE	t	p	Lower 95% CI	Upper 95% CI
AR(1)	0.80	0.04	22.15	<0.0001	0.73	0.87
Accounts suspended	0.00	0.01	-0.14	0.89	-0.02	0.02
Content removed	-0.02	0.02	-1.11	0.27	-0.05	0.01
African News	-0.94	0.45	-2.09	0.04	-1.83	-0.06
Malaysian News	-0.95	0.63	-1.51	0.13	-2.20	0.29
South African News	-0.72	0.67	-1.08	0.28	-2.03	0.59
Philippine News	-0.95	0.81	-1.18	0.24	-2.54	0.63
Canadian Vaccine Promotion	-0.64	0.39	-1.61	0.11	-1.41	0.14
UK Vaccine Promotion	0.04	0.54	0.08	0.94	-1.02	1.10
US Vaccine Promotion	-0.40	0.47	-0.85	0.39	-1.32	0.52
Vaccine Promoting Doctors	-0.55	0.34	-1.65	0.10	-1.22	0.11
Vaccine Promoting News	-0.13	0.27	-0.47	0.64	-0.65	0.40
Global Orgs.	-0.81	0.39	-2.09	0.04	-1.57	-0.05
Health & Science	-0.35	0.31	-1.14	0.26	-0.95	0.25
Canadian Vaccine Skeptics	-1.19	0.27	-4.34	<0.0001	-1.72	-0.65
UK Vaccine Skeptics	-0.66	0.50	-1.33	0.18	-1.64	0.32
Prominent US Skeptics	-0.53	0.27	-1.95	0.05	-1.06	0.00
Vaccine Skeptical Doctors	-1.21	0.29	-4.24	<0.0001	-1.77	-0.65
Vaccine Skeptical News	-0.95	0.24	-3.89	<0.0001	-1.43	-0.47
Anti-Globalist	-1.03	0.55	-1.88	0.06	-2.11	0.05
US Left Commentators	0.09	0.59	0.14	0.89	-1.08	1.25
US Left Influencers	-0.18	0.54	-0.33	0.74	-1.25	0.89
US Left Leaders	0.01	0.41	0.03	0.97	-0.79	0.82

US Right Commentators	-0.91	0.34	-2.67	0.008	-1.58	-0.24
US Right Influencers	-1.08	0.35	-3.05	0.002	-1.77	-0.38
US Right Leaders	0.46	0.48	0.96	0.34	-0.48	1.40
Indian Left	-0.57	1.00	-0.57	0.57	-2.53	1.38
Indian Right	-0.44	0.96	-0.46	0.65	-2.32	1.44
African News x Accounts suspended	-0.06	0.06	-1.03	0.30	-0.18	0.06
African News x Content removed	0.07	0.08	0.94	0.35	-0.08	0.22
Malaysian News x Accounts suspended	-0.07	0.10	-0.74	0.46	-0.26	0.12
Malaysian News x Content removed	0.05	0.10	0.54	0.59	-0.15	0.26
South African News x Accounts suspended	0.08	0.05	1.45	0.15	-0.03	0.18
South African News x Content removed	-0.06	0.09	-0.68	0.50	-0.23	0.11
Philippine News x Accounts suspended	-0.05	0.06	-0.87	0.39	-0.17	0.07
Philippine News x Content removed	0.04	0.10	0.39	0.70	-0.16	0.23
Canadian Vaccine Promotion x Accounts suspended	-0.03	0.04	-0.77	0.44	-0.11	0.05
Canadian Vaccine Promotion x Content removed	0.04	0.05	0.82	0.41	-0.05	0.13
UK Vaccine Promotion x Accounts suspended	0.01	0.05	0.27	0.79	-0.09	0.11
UK Vaccine Promotion x Content removed	-0.08	0.08	-0.91	0.37	-0.24	0.09
US Vaccine Promotion x Accounts suspended	-0.07	0.03	-2.14	0.03	-0.13	-0.01
US Vaccine Promotion x Content removed	0.01	0.06	0.18	0.86	-0.10	0.12
Vaccine Promoting Doctors x Accounts suspended	0.01	0.02	0.56	0.58	-0.03	0.06
Vaccine Promoting Doctors x Content removed	-0.01	0.04	-0.21	0.83	-0.08	0.06
Vaccine Promoting News x Accounts suspended	-0.09	0.03	-2.89	0.004	-0.15	-0.03
Vaccine Promoting News x Content removed	0.00	0.03	0.12	0.90	-0.06	0.07
Global Orgs. x Accounts suspended	-0.07	0.05	-1.45	0.15	-0.17	0.03
Global Orgs. x Content removed	0.05	0.07	0.67	0.50	-0.09	0.18
Health & Science x Accounts suspended	-0.03	0.02	-1.15	0.25	-0.07	0.02

Health & Science x Content removed	0.00	0.04	-0.04	0.97	-0.08	0.08
Canadian Vaccine Skeptics x Accounts suspended	0.16	0.03	4.76	<0.0001	0.09	0.22
Canadian Vaccine Skeptics x Content removed	0.00	0.03	-0.09	0.93	-0.07	0.06
UK Vaccine Skeptics x Accounts suspended	0.02	0.04	0.68	0.50	-0.05	0.10
UK Vaccine Skeptics x Content removed	0.03	0.05	0.59	0.56	-0.07	0.13
Prominent US Skeptics x Accounts suspended	0.02	0.03	0.75	0.46	-0.03	0.08
Prominent US Skeptics x Content removed	-0.02	0.03	-0.82	0.41	-0.08	0.03
Vaccine Skeptical Doctors x Accounts suspended	0.10	0.03	3.31	0.00	0.04	0.16
Vaccine Skeptical Doctors x Content removed	0.04	0.03	1.63	0.11	-0.01	0.10
Vaccine Skeptical News x Accounts suspended	0.01	0.02	0.60	0.55	-0.03	0.06
Vaccine Skeptical News x Content removed	0.04	0.03	1.26	0.21	-0.02	0.10
Anti-Globalist x Accounts suspended	0.14	0.05	2.58	0.01	0.03	0.24
Anti-Globalist x Content removed	-0.06	0.08	-0.74	0.46	-0.22	0.10
US Left Commentators x Accounts suspended	-0.13	0.05	-2.45	0.02	-0.23	-0.03
US Left Commentators x Content removed	0.04	0.06	0.70	0.49	-0.07	0.15
US Left Influencers x Accounts suspended	-0.10	0.03	-3.20	0.001	-0.17	-0.04
US Left Influencers x Content removed	0.03	0.07	0.44	0.66	-0.10	0.16
US Left Leaders x Accounts suspended	-0.08	0.04	-2.15	0.03	-0.15	-0.01
US Left Leaders x Content removed	-0.04	0.04	-0.95	0.34	-0.12	0.04
US Right Commentators x Accounts suspended	0.03	0.03	0.89	0.37	-0.04	0.10
US Right Commentators x Content removed	0.04	0.05	0.79	0.43	-0.06	0.14
US Right Influencers x Accounts suspended	0.01	0.04	0.35	0.73	-0.06	0.09
US Right Influencers x Content removed	0.10	0.05	1.85	0.07	-0.01	0.20
Indian Left x Accounts suspended	-0.07	0.12	-0.58	0.56	-0.30	0.16
Indian Left x Content removed	0.03	0.12	0.22	0.83	-0.22	0.27
US Right Leaders x Accounts suspended	-0.04	0.07	-0.60	0.55	-0.17	0.09

US Right Leaders x Content removed	-0.13	0.09	-1.47	0.14	-0.31	0.04
Indian Right x Accounts suspended	-0.03	0.10	-0.31	0.76	-0.22	0.16
Indian Right x Content removed	0.00	0.15	0.00	1.00	-0.30	0.30

Note. B = regression coefficient. SE = Standard Error. CI = Confidence Interval. AR(1) = Autoregressive term of order 1.

Table S9: LSDV regression table predicting log-transformed retweets per follower

	B	SE	t	p	Lower 95% CI	Upper 95% CI
AR(1)	0.57	0.06	9.82	<0.001	0.46	0.69
Accounts suspended	0.00	0.01	-0.25	0.81	-0.02	0.01
Content removed	0.00	0.01	0.33	0.74	-0.01	0.02
African News	-3.36	0.50	-6.69	<0.001	-4.35	-2.37
Malaysian News	-3.48	0.69	-5.02	<0.001	-4.85	-2.12
South African News	-3.14	0.83	-3.78	<0.001	-4.78	-1.51
Philippine News	-4.22	0.71	-5.98	<0.001	-5.61	-2.84
Canadian Vaccine Promotion	-3.32	0.47	-7.04	<0.001	-4.24	-2.39
UK Vaccine Promotion	-2.84	0.42	-6.82	<0.001	-3.66	-2.03
US Vaccine Promotion	-3.94	0.50	-7.82	<0.001	-4.93	-2.95
Vaccine Promoting Doctors	-2.54	0.39	-6.47	<0.001	-3.31	-1.77
Vaccine Promoting News	-3.42	0.54	-6.31	<0.001	-4.48	-2.35
Global Orgs.	-3.62	0.52	-6.97	<0.001	-4.64	-2.60
Health & Science	-3.18	0.46	-6.97	<0.001	-4.08	-2.29
Canadian Vaccine Skeptics	-2.95	0.41	-7.15	<0.001	-3.76	-2.14
UK Vaccine Skeptics	-2.70	0.38	-7.13	<0.001	-3.44	-1.96

Prominent US Skeptics	-2.92	0.41	-7.18	<0.001	-3.72	-2.12
Vaccine Skeptical Doctors	-2.87	0.39	-7.34	<0.001	-3.64	-2.10
Vaccine Skeptical News	-3.31	0.49	-6.78	<0.001	-4.27	-2.35
Anti-Globalist	-2.93	0.49	-5.93	<0.001	-3.90	-1.96
US Left Commentators	-3.05	0.43	-7.07	<0.001	-3.90	-2.21
US Left Influencers	-2.76	0.41	-6.79	<0.001	-3.56	-1.96
US Left Leaders	-3.20	0.50	-6.39	<0.001	-4.18	-2.22
US Right Commentators	-3.35	0.47	-7.18	<0.001	-4.27	-2.44
US Right Influencers	-3.27	0.55	-5.98	<0.001	-4.34	-2.20
US Right Leaders	-3.01	0.47	-6.42	<0.001	-3.93	-2.09
Indian Left	-3.58	0.60	-5.99	<0.001	-4.76	-2.41
Indian Right	-3.06	0.58	-5.27	<0.001	-4.20	-1.92
African News X Accounts suspended	-0.01	0.04	-0.13	0.90	-0.09	0.08
African News X Content removed	0.01	0.05	0.20	0.84	-0.08	0.10
Malaysian News X Accounts suspended	0.04	0.05	0.74	0.46	-0.06	0.14
Malaysian News X Content removed	0.01	0.07	0.15	0.88	-0.12	0.14
South African News X Accounts suspended	0.12	0.07	1.64	0.10	-0.02	0.27
South African News X Content removed	-0.08	0.10	-0.75	0.45	-0.28	0.13
Philippine News X Accounts suspended	-0.01	0.05	-0.28	0.78	-0.12	0.09
Philippine News X Content removed	0.06	0.06	1.07	0.29	-0.05	0.18
Canadian Vaccine Promotion X Accounts suspended	0.06	0.03	2.56	0.01	0.02	0.11
Canadian Vaccine Promotion X Content removed	0.00	0.04	0.04	0.97	-0.07	0.07
UK Vaccine Promotion X Accounts suspended	0.00	0.02	0.14	0.89	-0.03	0.03
UK Vaccine Promotion X Content removed	-0.02	0.02	-1.57	0.12	-0.05	0.01
US Vaccine Promotion X Accounts suspended	0.05	0.03	1.56	0.12	-0.01	0.10
US Vaccine Promotion X Content removed	0.07	0.03	2.61	0.009	0.02	0.12

Vaccine Promoting Doctors X Accounts suspended	0.02	0.02	1.02	0.31	-0.02	0.05
Vaccine Promoting Doctors X Content removed	-0.02	0.02	-1.20	0.23	-0.06	0.02
Vaccine Promoting News X Accounts suspended	0.02	0.04	0.51	0.61	-0.06	0.10
Vaccine Promoting News X Content removed	0.00	0.04	-0.01	0.99	-0.09	0.09
Global Orgs. X Accounts suspended	0.04	0.02	2.21	0.03	0.01	0.08
Global Orgs. X Content removed	0.03	0.03	0.86	0.39	-0.03	0.09
Health & Science X Accounts suspended	0.02	0.02	1.01	0.32	-0.02	0.06
Health & Science X Content removed	0.01	0.02	0.60	0.55	-0.03	0.05
Canadian Vaccine Skeptics X Accounts suspended	0.09	0.02	4.07	<0.001	0.05	0.13
Canadian Vaccine Skeptics X Content removed	0.03	0.02	1.92	0.06	0.00	0.07
UK Vaccine Skeptics X Accounts suspended	-0.01	0.01	-1.73	0.08	-0.03	0.00
UK Vaccine Skeptics X Content removed	0.04	0.01	3.85	<0.001	0.02	0.07
Prominent US Skeptics X Accounts suspended	0.02	0.01	1.89	0.06	0.00	0.04
Prominent US Skeptics X Content removed	0.05	0.02	3.13	0.002	0.02	0.08
Vaccine Skeptical Doctors X Accounts suspended	-0.01	0.01	-0.56	0.58	-0.04	0.02
Vaccine Skeptical Doctors X Content removed	0.09	0.02	5.35	<0.001	0.05	0.12
Vaccine Skeptical News X Accounts suspended	0.07	0.03	2.50	0.01	0.01	0.12
Vaccine Skeptical News X Content removed	0.05	0.03	1.96	0.05	0.00	0.11
Anti-Globalist X Accounts suspended	0.11	0.02	4.59	<0.001	0.06	0.16
Anti-Globalist X Content removed	0.04	0.04	0.99	0.32	-0.04	0.12
US Left Commentators X Accounts suspended	-0.01	0.01	-0.69	0.49	-0.03	0.02
US Left Commentators X Content removed	0.02	0.02	1.21	0.23	-0.01	0.05
US Left Influencers X Accounts suspended	-0.03	0.02	-1.84	0.07	-0.06	0.00
US Left Influencers X Content removed	0.01	0.02	0.88	0.38	-0.02	0.04
US Left Leaders X Accounts suspended	0.03	0.04	0.74	0.46	-0.05	0.10
US Left Leaders X Content removed	0.00	0.05	-0.07	0.95	-0.10	0.09

US Right Commentators X Accounts suspended	0.04	0.02	2.12	0.03	0.00	0.08
US Right Commentators X Content removed	0.09	0.02	4.39	<0.001	0.05	0.13
US Right Influencers X Accounts suspended	0.01	0.02	0.70	0.49	-0.03	0.05
US Right Influencers X Content removed	0.07	0.04	1.89	0.06	0.00	0.15
US Right Leaders X Accounts suspended	0.02	0.02	0.83	0.41	-0.03	0.07
US Right Leaders X Content removed	0.01	0.03	0.18	0.86	-0.05	0.06
Indian Left X Accounts suspended	0.04	0.04	1.16	0.25	-0.03	0.11
Indian Left X Content removed	0.06	0.05	1.32	0.19	-0.03	0.16
Indian Right X Accounts suspended	-0.17	0.06	-2.64	0.009	-0.29	-0.04
Indian Right X Content removed	0.09	0.08	1.11	0.27	-0.07	0.25

Note. B = regression coefficient. SE = Standard Error. CI = Confidence Interval. AR(1) = Autoregressive term of order 1.

Table S10: LSDV regression table predicting changes in URL domain quality

	B	SE	t	p	Lower 95% CI	Upper 95% CI
AR(1)	-0.01	0.01	-2.38	0.02	-0.02	0.00
Accounts suspended	0.00	0.00	-0.41	0.68	0.00	0.00
Content removed	0.00	0.00	0.78	0.44	0.00	0.00
African News	0.62	0.07	8.38	<0.001	0.47	0.77
Malaysian News	0.64	0.08	7.96	<0.001	0.49	0.80
South African News	0.58	0.10	5.65	<0.001	0.38	0.79
Philippine News	0.59	0.06	10.54	<0.001	0.48	0.71
CA Vaccine Promotion	0.68	0.04	15.89	<0.001	0.59	0.76
UK Vaccine Promotion	0.64	0.04	14.89	<0.001	0.56	0.73

US Vaccine Promotion	0.65	0.05	13.99	<0.001	0.56	0.75
Vaccine Promoting Doctors	0.69	0.04	19.02	<0.001	0.62	0.77
Vaccine Promoting News	0.66	0.05	12.81	<0.001	0.56	0.76
Global Orgs.	0.66	0.05	12.41	<0.001	0.56	0.77
Health & Science	0.69	0.04	15.72	<0.001	0.61	0.78
Canadian Vaccine Skeptics	0.48	0.07	6.78	<0.001	0.34	0.62
UK Vaccine Skeptics	0.44	0.05	9.94	<0.001	0.36	0.53
Prominent US Skeptics	0.46	0.05	9.00	<0.001	0.36	0.56
Vaccine Skeptical Doctors	0.53	0.06	9.64	<0.001	0.43	0.64
Vaccine Skeptical News	0.61	0.08	7.42	<0.001	0.45	0.78
Anti-Globalist	0.46	0.07	7.05	<0.001	0.33	0.59
US Left Commentators	0.68	0.05	14.00	<0.001	0.59	0.78
US Left Influencers	0.67	0.04	16.17	<0.001	0.59	0.75
US Left Leaders	0.67	0.04	15.17	<0.001	0.58	0.76
US Right Commentators	0.52	0.07	7.46	<0.001	0.38	0.66
US Right Influencers	0.48	0.06	7.84	<0.001	0.36	0.59
US Right Leaders	0.41	0.06	6.82	<0.001	0.29	0.53
Indian Left	0.64	0.07	9.26	<0.001	0.51	0.78
Indian Right	0.56	0.05	10.49	<0.001	0.45	0.66
African News X Accounts suspended	-0.01	0.01	-2.19	0.03	-0.02	0.00
African News X Content removed	0.01	0.01	1.32	0.19	-0.01	0.03
Malaysian News X Accounts suspended	-0.03	0.01	-3.12	0.002	-0.04	-0.01
Malaysian News X Content removed	0.01	0.01	1.61	0.11	0.00	0.03
South African News X Accounts suspended	-0.03	0.01	-3.41	0.001	-0.05	-0.01
South African News X Content removed	0.02	0.01	1.42	0.16	-0.01	0.04
Philippine News X Accounts suspended	-0.01	0.00	-1.82	0.07	-0.01	0.00

Philippine News X Content removed	0.00	0.01	0.92	0.36	-0.01	0.01
Canadian Vaccine Promotion X Accounts suspended	0.00	0.00	1.61	0.11	0.00	0.00
Canadian Vaccine Promotion X Content removed	0.00	0.00	2.07	0.04	0.00	0.01
UK Vaccine Promotion X Accounts suspended	0.00	0.00	1.19	0.23	0.00	0.01
UK Vaccine Promotion X Content removed	0.00	0.00	-0.53	0.60	-0.01	0.00
US Vaccine Promotion X Accounts suspended	0.00	0.00	0.44	0.66	0.00	0.00
US Vaccine Promotion X Content removed	0.00	0.00	0.62	0.54	0.00	0.00
Vaccine Promoting Doctors X Accounts suspended	0.00	0.00	0.75	0.45	0.00	0.01
Vaccine Promoting Doctors X Content removed	0.00	0.00	0.13	0.90	0.00	0.00
Vaccine Promoting News X Accounts suspended	0.00	0.00	-1.30	0.20	-0.01	0.00
Vaccine Promoting News X Content removed	0.00	0.00	0.91	0.37	0.00	0.01
Global Orgs. X Accounts suspended	-0.01	0.00	-2.42	0.02	-0.02	0.00
Global Orgs. X Content removed	0.01	0.01	1.07	0.29	0.00	0.02
Health & Science X Accounts suspended	0.00	0.00	2.57	0.01	0.00	0.01
Health & Science X Content removed	0.00	0.00	0.46	0.65	0.00	0.00
Canadian Vaccine Skeptics X Accounts suspended	-0.03	0.01	-3.24	0.001	-0.04	-0.01
Canadian Vaccine Skeptics X Content removed	0.02	0.01	2.40	0.02	0.00	0.04
UK Vaccine Skeptics X Accounts suspended	-0.01	0.01	-1.31	0.19	-0.02	0.00
UK Vaccine Skeptics X Content removed	0.00	0.00	1.07	0.29	0.00	0.01
Prominent US Skeptics X Accounts suspended	-0.02	0.01	-3.91	<0.001	-0.03	-0.01
Prominent US Skeptics X Content removed	0.00	0.01	0.77	0.44	-0.01	0.02
Vaccine Skeptical Doctors X Accounts suspended	-0.02	0.01	-2.57	0.01	-0.03	0.00
Vaccine Skeptical Doctors X Content removed	0.01	0.01	0.95	0.34	-0.01	0.02
Vaccine Skeptical News X Accounts suspended	-0.02	0.01	-2.69	0.007	-0.04	-0.01
Vaccine Skeptical News X Content removed	0.00	0.01	-0.28	0.78	-0.02	0.02
Anti-Globalist X Accounts suspended	-0.01	0.01	-1.53	0.13	-0.02	0.00

Anti-Globalist X Content removed	0.00	0.01	0.63	0.53	-0.01	0.02
US Left Commentators X Accounts suspended	0.00	0.00	0.74	0.46	0.00	0.01
US Left Commentators X Content removed	0.00	0.00	-0.99	0.32	-0.01	0.00
US Left Influencers X Accounts suspended	0.00	0.00	-0.12	0.90	-0.01	0.01
US Left Influencers X Content removed	0.00	0.00	0.69	0.49	0.00	0.01
US Left Leaders X Accounts suspended	0.00	0.00	-1.42	0.16	-0.01	0.00
US Left Leaders X Content removed	0.00	0.00	0.45	0.66	-0.01	0.01
US Right Commentators X Accounts suspended	-0.02	0.01	-2.94	0.003	-0.03	-0.01
US Right Commentators X Content removed	0.01	0.01	1.14	0.26	-0.01	0.03
US Right Influencers X Accounts suspended	-0.01	0.01	-2.55	0.01	-0.02	0.00
US Right Influencers X Content removed	0.00	0.01	-0.44	0.66	-0.02	0.01
US Right Leaders X Accounts suspended	-0.01	0.01	-2.37	0.02	-0.02	0.00
US Right Leaders X Content removed	0.00	0.01	0.80	0.42	-0.01	0.02
Indian Left X Accounts suspended	-0.03	0.01	-4.64	<0.001	-0.05	-0.02
Indian Left X Content removed	0.01	0.01	1.46	0.15	0.00	0.03
Indian Right X Accounts suspended	-0.01	0.00	-3.31	0.001	-0.02	-0.01
Indian Right X Content removed	0.00	0.00	1.05	0.29	0.00	0.01

Note. B = regression coefficient. SE = Standard Error. CI = Confidence Interval. AR(1) = Autoregressive term of order 1.

Table S11: CITS results comparing US political right-wing clusters to left-wing comparators. Results include content proportions, retweets per follower, and URL domain quality ratings during the Content Removal and Five Strikes policy regimes.

		Content Proportions											
		Content Removal				Five Strikes				Reinstatement			
			95% CI				95% CI				95% CI		
		OR	Low	Hi	p	OR	Low	Hi	p	OR	Low	Hi	p
Leaders	Left	1.58	1.37	1.83	<0.001	0.46	0.44	0.48	<0.001	0.94	0.67	1.31	0.35
	Right	0.14	0.11	0.17	<0.001	0.36	0.33	0.38	<0.001	1.85	1.32	2.58	<0.001
	Difference	0.09	0.07	0.11	<0.001	0.78	0.72	0.84	<0.001	1.97	1.23	3.17	0.003
Influencers	Left	0.92	0.66	1.28	0.31	0.49	0.44	0.53	<0.001	0.83	0.38	1.81	0.32
	Right	0.98	0.79	1.21	0.41	3.58	3.34	3.83	<0.001	1.29	0.84	1.99	0.12
	Difference	1.06	0.72	1.58	0.38	7.37	6.55	8.30	<0.001	1.55	0.64	3.77	0.17
Commentators	Left	0.73	0.57	0.94	0.007	0.81	0.76	0.87	<0.001	1.27	0.67	2.41	0.23
	Right	0.92	0.81	1.05	0.10	2.74	2.64	2.84	<0.001	0.90	0.66	1.24	0.26
	Difference	1.26	0.95	1.67	0.05	3.38	3.12	3.65	<0.001	0.71	0.35	1.44	0.17
		Retweets per Follower											
		RR	Low	Hi	p	RR	Low	Hi	p	RR	Low	Hi	p
Leaders	Left	0.46	0.36	0.60	<0.001	1.61	1.50	1.73	<0.001	1.38	1.04	1.82	0.01
	Right	1.05	0.96	1.15	0.16	1.16	1.13	1.20	<0.001	1.11	0.89	1.39	0.17
	Difference	2.25	1.73	2.94	<0.001	0.72	0.67	0.78	<0.001	0.81	0.57	1.16	0.13
Influencers	Left	0.91	0.83	1.00	0.03	0.93	0.90	0.95	<0.001	1.89	1.69	2.11	<0.001
	Right	1.70	1.54	1.89	<0.001	1.12	1.09	1.15	<0.001	0.91	0.74	1.11	0.18

	Difference	1.87	1.63	2.15	<0.001	1.21	1.17	1.26	<0.001	0.48	0.38	0.61	<0.001
Commentators	Left	0.92	0.84	1.00	0.03	1.06	1.03	1.09	<0.001	0.97	0.84	1.11	0.31
	Right	1.30	1.20	1.41	<0.001	1.88	1.84	1.92	<0.001	0.95	0.81	1.12	0.27
	Difference	1.42	1.26	1.60	<0.001	1.77	1.71	1.83	<0.001	0.98	0.79	1.22	0.44
							Domain Quality						
		Δ	Low	Hi	p	Δ	Low	Hi	p	Δ	Low	Hi	p
Leaders	Left	0.00	-0.01	0.01	0.46	-0.01	-0.02	-0.01	<0.001	-0.04	-0.06	-0.02	<0.001
	Right	0.01	-0.02	0.05	0.24	-0.04	-0.05	-0.03	<0.001	0.02	-0.02	0.07	0.15
	Difference	0.01	-0.02	0.05	0.24	-0.03	-0.04	-0.02	<0.001	0.06	0.01	0.11	0.007
Influencers	Left	0.00	-0.02	0.02	0.46	0.00	-0.01	0.00	0.05	0.00	-0.02	0.03	0.38
	Right	0.00	-0.04	0.04	0.48	-0.04	-0.05	-0.03	<0.001	-0.02	-0.06	0.02	0.17
	Difference	0.00	-0.05	0.05	0.50	-0.04	-0.05	-0.03	<0.001	-0.02	-0.07	0.02	0.16
Commentators	Left	0.02	0.01	0.04	0.004	-0.01	-0.01	-0.01	<0.001	-0.03	-0.17	0.11	0.31
	Right	0.02	-0.02	0.05	0.19	-0.07	-0.08	-0.06	<0.001	-0.05	-0.21	0.11	0.27
	Difference	-0.01	-0.05	0.03	0.33	-0.06	-0.07	-0.05	<0.001	-0.02	-0.23	0.20	0.44
				% Topics Likely Violating Twitter's Medical Misinformation Policies									
		OR	Low	Hi	p	OR	Low	Hi	p	OR	Low	Hi	p
Leaders	Left	0.74	0.59	0.94	0.01	1.00	0.94	1.07	0.97	0.72	0.47	1.11	0.14
	Right	0.92	0.65	1.31	0.66	0.89	0.81	0.97	0.01	1.14	0.74	1.75	0.56
	Difference	1.25	0.82	1.90	0.31	0.89	0.79	0.99	0.04	1.57	0.85	2.88	0.15
Influencers	Left	0.73	0.49	1.08	0.11	1.04	0.94	1.15	0.48	1.17	0.67	2.05	0.57
	Right	0.86	0.62	1.19	0.36	1.12	1.02	1.22	0.02	1.09	0.66	1.82	0.74
	Difference	1.18	0.71	1.96	0.52	1.08	0.94	1.24	0.29	0.93	0.44	1.98	0.85
Commentators	Left	0.71	0.51	1.00	0.05	1.29	1.18	1.41	< 0.001	1.04	0.57	1.88	0.91
	Right	0.95	0.79	1.14	0.56	1.40	1.33	1.47	< 0.001	1.33	0.91	1.93	0.14
	Difference	1.33	0.90	1.95	0.15	1.08	0.98	1.20	0.14	1.28	0.63	2.59	0.49

		% Topics Containing Commentary											
		OR	Low	Hi	p	OR	Low	Hi	p	OR	Low	Hi	p
Leaders	Left	1.02	0.66	1.58	0.93	0.72	0.64	0.81	< 0.001	1.44	0.93	2.24	0.11
	Right	1.14	0.82	1.58	0.44	1.21	1.10	1.33	< 0.001	0.96	0.60	1.53	0.87
	Difference	1.12	0.64	1.93	0.69	1.69	1.45	1.96	< 0.001	0.67	0.35	1.27	0.22
Influencers	Left	1.04	0.58	1.84	0.90	1.25	1.09	1.43	0.001	1.63	0.95	2.79	0.08
	Right	1.43	0.87	2.33	0.15	1.26	1.07	1.47	0.005	0.78	0.47	1.29	0.34
	Difference	1.38	0.65	2.93	0.40	1.01	0.82	1.24	0.93	0.48	0.23	1.01	0.05
Commentators	Left	1.21	0.84	1.73	0.31	0.62	0.56	0.68	< 0.001	1.22	0.76	1.97	0.40
	Right	1.13	0.77	1.67	0.52	0.97	0.88	1.08	0.62	0.86	0.55	1.32	0.48
	Difference	0.94	0.55	1.60	0.82	1.57	1.36	1.81	< 0.001	0.70	0.37	1.33	0.28
		% Pro-Vaccine Topics											
		OR	Low	Hi	p	OR	Low	Hi	p	OR	Low	Hi	p
Leaders	Left	1.71	1.09	2.71	0.02	1.23	1.07	1.40	0.003	0.61	0.36	1.02	0.06
	Right	1.07	0.83	1.38	0.61	1.04	0.97	1.12	0.23	1.15	0.83	1.60	0.41
	Difference	0.62	0.37	1.05	0.08	0.85	0.73	0.99	0.04	1.90	1.03	3.50	0.04
Influencers	Left	1.40	1.04	1.90	0.03	0.64	0.58	0.69	< 0.001	1.10	0.67	1.81	0.71
	Right	0.93	0.73	1.20	0.58	0.78	0.72	0.84	< 0.001	1.20	0.81	1.78	0.37
	Difference	0.66	0.45	0.98	0.04	1.22	1.09	1.36	< 0.001	1.09	0.58	2.06	0.79
Commentators	Left	1.68	1.22	2.33	0.002	1.02	0.88	1.17	0.83	0.69	0.40	1.20	0.19
	Right	1.08	0.89	1.30	0.43	0.58	0.55	0.61	< 0.001	1.03	0.75	1.43	0.85
	Difference	0.64	0.44	0.93	0.02	0.57	0.49	0.66	< 0.001	1.49	0.79	2.81	0.22

Note. OR = Odds Ratio. RR = Risk Ratio. Δ = difference.

Table S12: Twitter communities extracted using Louvain algorithm

	Most Active Accounts<br>Most Followers				
Cluster Name	1	2	3	4	5
			Vaccine Skeptics		
Canadian Vaccine Skeptics	PaulMitchell_AB	MaximeBernier	MelissaLMRogers	JamieSale	TheRealKeean
	nationalpost	PierrePoilievre	TheTorontoSun	jkenney	ezralevant
Vaccine Skeptical Doctors	RWMaloneMD	AlexBerenson	MichaelPSenger	ianmSC	MartinKulldorff
	bmj_latest	BretWeinstein	AlexBerenson	thierrybaudet	PierreKory
Anti-Globalist	DrEliDavid	drsimonegold	Rob_Roos	PrisonPlanet	TuckerCarlson
	TuckerCarlson	Cobratate	PrisonPlanet	TulsiGabbard	AnaPaulaVolei
Vaccine Skeptical News	disclosetv	zerohedge	Breaking911	nypost	TheInsiderPaper
	nypost	theRealKiyosaki	zerohedge	OscarDeLaHoya	Breaking911
UK Vaccine Skeptics	BernieSpofforth	TonyHinton2016	EssexPR	JamesMelville	toadmeister
	Telegraph	Nigel_Farage	RealDeniseWelch	ProfKarolSikora	JuliaHB1
Prominent US Skeptics	RobertKennedyJr	HighWireTalk	o_rips	uTobian	BusyDrT
	RobSchneider	mercola	RobertKennedyJr	davidicke	delbigtree
			Vaccine Promotion		
Canadian Vaccine Promotion	BogochIsaac	CTVNews	CP24	JustinTrudeau	picardonhealth
	JustinTrudeau	CBCNews	CP24	globeandmail	CBCAlerts
Vaccine Promoting Doctors	DrEricDing	DrTomFrieden	PeterHotez	Craig_A_Spencer	Cleavon_MD
	AndrewYang	mehdirhasan	Surgeon_General	propublica	theintercept
Global Orgs.	UNICEF	WHO	DrTedros	setiogi	vonderleyen
	selenagomez	MileyCyrus	billboard	WHO	UNICEF

Vaccine Promotion News	nytimes	CNN	business	AP	ABC
	cnnbrk	CNN	nytimes	BBCBreaking	BBCWorld
Health & Science	EricTopol	HelenBranswell	ashishkjha	MaxCRoser	megtirrell
	sciam	NateSilver538	ScienceMagazine	NaturePortfolio	Nature
UK Vaccine Promotion	NHSuk	DHSCgovuk	doctor_oxford	chrischirp	devisridhar
	piersmorgan	10DowningStreet	IanMcKellen	BorisJohnson	ProfBrianCox
US Vaccine Promotion	CDCgov	HHSGov	US_FDA	AZDHS	WebMD
	CDCgov	WebMD	MayoClinic	CDCemergency	ClevelandClinic
United States Left-Wing Politics					
US Left Commentators	kylegriffin1	OccupyDemocrats	joncoopertweets	AnaCabrera	ASlavitt
	maddow	SenWarren	MSNBC	jaketapper	GeorgeTakei
US Left Influencers	sailorrooscout	dcmadness202	MurjaniRawls	___inCANdescent	taratesoro
	RealHughJackman	AOC	SenSanders	BernieSanders	LilNasX
US Left Leaders	POTUS	JoeBiden	WhiteHouse	GavinNewsom	VP
	BarackObama	JoeBiden	NBA	POTUS	MichelleObama
United States Right-Wing Politics					
US Right Commentators	RepThomasMassie	ClayTravis	SharylAttkisson	Timcast	JesseKellyDC
	benshapiro	stoolpresidente	drdrew	megynkelly	jordanbpeterson
US Right Influencers	ChuckCallesto	RealCandaceO	catturd2	JackPosobiec	Jim_Jordan
	FoxNews	DonaldJTrumpJr	seanhannity	tedcruz	RandPaul
US Right Leaders	Thomas1774Paine	WhiteHouse	realDonaldTrump	RealJamesWoods	Mike_Pence
	realDonaldTrump	WhiteHouse	IvankaTrump	Mike_Pence	marcorubio
Other					
African News	spectatorindex	yodifiji	AfricaFactsZone	DrOlufunmilayo	NphcdaNG
	elonmusk	premierleague	SkySportsNews	channelstv	MobilePunch
Malaysian News	Khairykj	HausofHilton	DGHisham	NewsBFM	Medguy1Sam

	Khairykj	501Awani	staronline	KKMPutrajaya	malaysiakini
Philippine News	ABSCBNNews	inquirerdotnet	cnnphilippines	rapplerdotcom	gmanews
	ABSCBNNews	gmanews	ANCALERTS	lizasoberano	iamkarendavila
South African News	News24	eNCA	miamalan	SAfmnews	SizweLo
	News24	eNCA	CyrilRamaphosa	SABCNews	PresidencyZA
Indian Right	narendramodi	ANI	AskAnshul	OpIndia_com	NorbertElekes
	narendramodi	BillGates	PMOIndia	AmitShah	BJP4India
Indian Left	FaheemYounus	RahulGandhi	pbhushan1	ndtv	SaketGokhale
	ArvindKejriwal	RahulGandhi	ndtv	chetan_bhagat	sardesairajdeep

Supplementary Information

Simulation Study

We constructed a simulation, based on [1], to better articulate how a system's architecture can facilitate or inhibit a social media platform's control efforts. Social media platforms are very large, hosting hundreds of millions or even billions of daily active users. Directly measuring and modeling networks of this size is prohibitive. Furthermore, the specific network structure of any given social media platform is constantly changing. The aim of this simulation is to rank different platform architectures regarding the extent to which they might facilitate or inhibit control of information flow.

Since we cannot directly measure *orders of growth* of different social media platform architectures. Our aim in doing so is to be able to assess whether a platform whose design follows a given architecture is more or less flexible — and therefore controllable — than another platform following a different architecture. Our simulation is based on Moses' Theory of Generic System Architectures [1], which quantifies the *flexibility* of different structures by counting up the total number of paths in the corresponding network. Flexibility is important because Moses theorizes that it is inversely related to its controllability [2]. The rationale is that if individual paths or nodes are disrupted, e.g., because content or accounts are removed, other accounts on the same platform can still access interdicted information through the use of alternate pathways. When the nodes represent social media accounts that are operating in a manner that is not aligned with the platform owner (e.g., when anti-vaccine accounts post vaccine misinformation despite a platform's policy prohibiting this misinformation), this flexibility can be used to undermine the platform owner's control efforts because attempts to remove posts or accounts can be circumvented by relying on the same alternate paths that facilitate flexibility.

Ideally, we would simulate several networks with the aim of calculating each one's total number of alternate pathways through which information might flow. However, exhaustive enumeration of all paths in a graph is known to be a #P-complete problem [3, 4], meaning that this enumeration can only be calculated precisely by brute force. This is prohibitively computationally expensive for large graphs. We therefore approximate the total number of paths in a graph using its communicability [5] – a widely-used proxy that is more computationally tractable [6, 7, 8, 9]. Specifically, the total network

communicability $C^{TN}(A)$ for each graph can be calculated by computing the exponential of its adjacency matrix,

$$[e^{(A)}]_{i,j} = \sum_{k=1}^{\infty} \frac{[A^k]_{i,j}}{k!} \tag{1}$$

and then summing across all nodes.

$$C^{TN}(A) = \mathbf{1}^T(e^A)\mathbf{1} \tag{2}$$

Our approach is to estimate the *order of growth* of the total network communicability of networks associated with different social media platform architectures, and then to compare these across architectures. To do so, we simulated networks associated with several of the generic system architectures identified by Moses [1] varying the total number of nodes in each network. In practice, total network communicability follows similar orders of growth as those derived for number of paths in prior work [1].

Social Media Platform Architectures

We claim that Twitter's system architecture undermines its controllability. Beyond empirical evidence for this claim, the simulation in this section compares networks resembling Twitter's structure to networks with structures that result from other architectures.

Layered Hierarchies A platform's architecture may place constraints on how information is allowed to flow between accounts. One such constraint, used by Facebook [10], is known as "layering". In layered structures, nodes are divided into a set of layers arranged in a hierarchy such that nodes in layer n may only connect to nodes in layers $n-1$, n, or $n+1$. Furthermore, the internal structure of a layered hierarchy need not be specified [1]. Moses [1, 2, 11] theorizes that layering can make inflexible systems more flexible, but can also make overly-flexible (and hence uncontrollable) systems more controllable. For each architecture described below, we also examined how segmenting the resulting networks into two or three layers affected their communicability. Although this need not be the case in general, for the purposes of this simulation, we assume that each layer has the same structure. For example, a three-layered scale-free structure with 2400 nodes is made up of three scale-free networks with 800 nodes each, with connections between

nodes in adjacent layers. We examined the effects of segmenting each of the structures described below into 2- and 3-layered hierarchies, holding the total number of nodes constant.

Generic System Architectures

For purposes of comparison, we generated several network structures based on the generic system architectures defined in [1].

Regular Trees One of the most restrictive system architectures is a regular tree structure, which requires that each node has exactly one parent and all but the leaf nodes have a fixed number of children. Although social media platforms are never this restrictive in practice, platforms such as Reddit or online discussion boards have some tree-like properties such as a requirement that messages can only be seen by followers of a given subreddit and, in some cases, are subject to removal by a moderator. Thus, tree structures represent a limiting case.

We generated tree-structured graphs using the IGRAPH python package [12] examining values of the tree branching factors (number of children per parent node) ranging from 2 through 9. For each branching factor, we generated trees with depth ranging from 2 through 9 and retained all trees with at most 2400 nodes. We calculated the total network communicability for each network and found that, holding branching factor fixed, communicability grows linearly with the total number of nodes, similar to the relationship shown in prior work [1] (see Table S1).

Regular Lattices A somewhat less restrictive system architecture is a regular lattice structure, in which each node has exactly d neighbors. This structure is also quite unrealistic for social media platforms. However, what it has in common with Twitter is the absence of an explicit hierarchy. We therefore include it for comparison purposes.

We generated lattices using the IGRAPH python package [12] with the dimensionality (total number of neighbors) of each node ranging from 2 through 5 retaining all lattices with at most 2400 nodes. We calculated the total network communicability for each network and found that, holding lattice dimensionality fixed, communicability grows in an inverse power relationship with the total number of nodes per lattice dimension, as shown in prior work [1] (see Table S2). Since the number of nodes in the network is equal to the

number of nodes per dimension raised to the power of d, the overall order of growth of these lattices is linear, albeit higher than trees.

Regular Toruses A variant of the lattice structure is the regular torus, in which all edges of the lattice “wrap around”. This structure, although still unrealistic for real social media platforms, is nevertheless the starting point for the Watts-Strogatz model, which is intended to induce the small world effect after random rewiring [13]. We therefore include this model because it is an example of an architecture that induces community structure.

We generated toruses using the IGRAPH python package [12] with the dimensionality (total number of neighbors) of each node ranging from 2 through 5 retaining all lattices with at most 2400 nodes. We calculated the total network communicability for each network and found that, holding torus dimensionality fixed, communicability grows linearly with the total number of nodes in the torus (see Table S4) and, in practice, has very similar communicability values to those found in regular lattices for single-layered structures; however, as the number of layers increases toruses appear to be less flexible, and hence more controllable, than lattices.

Complete Graphs Trees, lattices, and toruses are all relatively sparse structures. In contrast, many social media clusters are strongly connected. An extreme version of this observation yields an architecture with a fully-connected “team” or “complete” structure, in which each node is connected to all of its neighbors.

We generated fully-connected graphs using the IGRAPH python package [12] with the total number of nodes ranging from 100 through 700 nodes in increments of 100 (larger numbers of nodes had communicability values that were so high that they led to an overflow error). We calculated the total network communicability for each network and found that communicability grows exponentially with the total number of nodes in the network (see Table S5).

Scale-Free Networks

Twitter does not impose any restrictions on which accounts may follow other accounts. Thus, it possesses an unrestricted architecture, which Moses [11] simply refers to as a “network”. Absent any restrictions, a significant body of empirical work has demonstrated that such networks tend to self-organize

into highly-clustered communities in a manner that approximates a scale-free network [14, 15, 16]. We therefore calculated the communicability of several scale free networks and compared the communicability of these structures to those resulting from "generic architectures" defined in prior work [1].

To generate scale free networks, we used the IGRAPH python package [12] to create graphs with degree distributions matching our data. Specifically, we fit a power law to the empirical degree distribution of our dataset using the POWERLAW python package [17], and estimated the power law exponent of this dataset as $\gamma = 1.46$. We next randomly generated a degree sequence following this power law degree distribution, and used this degree sequence to randomly generate a scale-free network. Given this network, we next introduced community structure into our network by generating 25 simulated communities, each of which were created using the Watts-Strogatz model [13] with an average degree of 1 and a rewiring probability of 0.10. Next, we randomly selected 50 pairs of nodes and added edges between them if they were not already adjacent and if they both had at least two edges.

We examined networks with 150, 300, 600, 1200, and 2400 nodes, simulating 1000 networks for each network size. For each simulated network, we calculated its total network communicability and extracted the median and 95% confidence bounds (i.e., the 2.5 and 97.5 percentile communicability values). We compared the communicability of these scale free networks to those associated with Moses' generic architectures (see Table S6).

Simulation results show that the total network communicability of scale-free networks grows as order $O(e^{\frac{n}{\gamma}})$, which is larger than all but the highest order of growth of all generic architectures. In contrast, platforms such as Facebook, which utilize layering, are likely more controllable given the same number of nodes. Although we leave a systematic exploration of multiple different platforms to future work, we observe that Reddit, which utilizes elements of tree-like structures, may among the most controllable of social media platforms.

Least Squares Dummy Variable (LSDV) analysis

Content Removal was Associated with Increased Virality of Vaccine Skeptical Content

We did not detect a significant relationship between pieces of content removed and overall content proportions in any vaccine skeptical community (perhaps due to reduced statistical power after aggregating the data monthly; see Table S8).

However, content removals were associated with increases in virality in several vaccine skeptical clusters and failed to significantly decrease virality in all vaccine skeptical clusters (see Table S9).

Finally, content removal seems to have been associated with a slight increase in URL domain quality among Canadian Vaccine Skeptics accounts Δ *= 0.02, 95% CI: 0.00 — 0.04,* $p = 0.006$, but had no detectable impact on the remaining clusters (see Table S10).

Account Removal Was Associated With More Content, More Virality, and Lower Information Quality

Despite reduced statistical power, we observed that content proportions increased as more accounts were removed in the Anti-Globalist, *OR = 1.14, 95% CI: 1.03 — 1.17,* $p = 0.006$, and Vaccine Skeptical Doctors clusters, *OR = 1.10, 95% CI: 1.04 — 1.17,* $p = 0.001$ (these odds ratios were calculated by exponentiating log-odds regression coefficients; see Table S8, Figure 2C). Account removals were also associated with increases in virality in several vaccine skeptical clusters and failed to significantly decrease virality in all vaccine skeptical clusters (see Table S9, Figure 2E). Finally, account removals were associated with decreases in information quality in most vaccine skeptical clusters (see Table S10 Figure 2G), including among Canadian Vaccine Skeptics, overcoming the positive association with content removals, Δ *= -0.03, 95% CI: -0.04 — -0.01,* $p < 0.001$.

ChatGPT Prompt for Topic Classification

You are a researcher helping classify Twitter discussion topics related to COVID vaccines.

Here are the top words for a topic: top_words

Here are some representative tweets from that topic: top_tweets

Please classify this topic into one of the following categories:

Antivaccine content:

1. Alternative Medicine: Content that promotes alternatives to vaccination or alternative or complementary health systems or critiques biomedicine; content that promotes the benefits of "natural" immunity.
2. Civil Liberties: Content that opposes vaccination as an infringement of personal liberty, including opposition to vaccine mandates, parental choice narratives, vaccines as government overreach, and fears of punishment related to nonvaccination.
3. Conspiracy: Content that presents specific conspiracy theories or conveys a broader "search for truth." Includes stories of fraud, cover-up, or collusion between government, doctors, and pharmaceutical companies; "rebel" spokespeople who speak "truth" at odds with the medical establishment; and unusual theories related to vaccines.
4. Morality: Content that opposes vaccination for specific ideological reasons. Includes religious beliefs or morally loaded topics.
5. Safety Concerns: Content that critiques the safety or effectiveness of vaccines. Includes notion that vaccines cause harm, injury, or death; that vaccines are toxic or contain poison; and that vaccines fail to provide immunity.

Provaccine content:

6. Pro-Science: Content that promotes vaccine science or science in general. Includes defending science against pseudoscientific claims.
7. Provaccine policy: Content supporting expanded vaccine policies. Includes support for mandatory vaccination, opposition to nonmedical vaccine exemptions, and opposition to "vaccine choice."

8. Criticizing antivaccine beliefs: Content focused on refuting antivaccine arguments or blaming/ shaming antivaccine individuals. Includes ideas that antivaxxers are uninformed, bad parents, endangering others, etc. Also includes aggressive appeals to vaccinate.
9. Promotion: Content focused on specific vaccine campaigns. Includes recommendations for vaccines, philanthropic vaccination campaigns, and details on when, where, or how to get a vaccine.
10. Safety and efficacy: Content that describes the vaccines as safe or effective. Includes successes of vaccines, and reduction in disease. Also includes benefits of vaccination or risks of nonvaccination.

Amplification strategies:

11. Retweets: Retweets make up majority of topic, suggesting organized retweeting effort (possibly bot-driven).
12. Hashtags: Use of common hashtags to promote content.
13. @mentions: Inserting @ to high-profile individuals and organizations to gain attention.
14. Politics: Engaging in partisan political debate, referencing political candidates.

15. Other

Also briefly explain your reasoning. Return the response in this format:
Category: <One of the 15 options>
Reason: <Your reasoning>

Annotation Procedures

We conducted several rounds of annotation to establish acceptable inter-rater reliabiltiy when labeling topics. In our initial round of anntation, two of the study's authors (WZ and AMJ) annotated a random sample of 611 topics — stratified by label — drawn from the set of topics for which ChatGPT produced unanimous agreement (there were no such topics in the "hashtags" and "@mentions" categories, and only 11 such topics in the "retweets" category) as "in violation" (including "mixed but mostly in violation", "commentary", "pro-vaccine" (including "mixed but mostly in pro-vaccine", or "other" (including "mixed with equal amounts pro- and anti-vaccine". Agreement between ChatGPT ratings and human annotators indicated an unacceptably low level of agreement, α = *0.50*, although this was comparable to agreement between human annotators without including ChatGPT ratings, α = *0.47*.

Two authors, AMJ and DAB, therefore conducted three rounds of twenty annotations each, discursively reconciled differences in coding, and developed a preliminary codebook before conducting a fourth round of twenty annotations. This fourth round yielded an acceptable level of agreement, α = *0.77*, which was comparable to agreement between human annotators without including ChatGPT ratings, α = *0.79*.

These results indicate that with a shared interpretation and clear definitions, both humans and the model were able to achieve acceptably high consistency. Having achieved high levels of agreement, we next undertook a larger wave of annotations with 300 samples, with results approaching an acceptable level of agreement, α = *0.66*, which was comparable to agreement between human annotators without including ChatGPT ratings, α = *0.65*.

This large-scale wave showed that even at scale, agreement between annotators remained within or near the range Krippendorff considers acceptable for drawing tentative conclusions [18]. We next refined the codebook and conducted a final wave of 300 annotations, yielded an acceptable level of agreement, α = *0.71*, which was comparable to agreement between human annotators without including ChatGPT ratings, α = *0.76*.

These final results represent the most ecologically valid assessment of our labeling process, reflecting production-scale conditions with refined definitions. All pairwise and three-way agreement levels in this round fall within the 0.67–0.80 range Krippendorff identifies as supporting tentative conclusions [18].

These results indicate that the annotation task is challenging but tractable.

Disagreements arise primarily from nuanced distinctions rather than random noise. Furthermore, model-human agreement is comparable to human-human agreement, especially after calibration and codebook refinement, supporting the use of these labels for analytic conclusions.

References

[1] Broniatowski, D. A. & Moses, J. Measuring flexibility, descriptive complexity, and rework potential in generic system architectures. *Systems Engineering* **19**, 207–221 (2016).

[2] Moses, J. *Flexibility and Its Relation to Complexity and Architecture*, 197–206 (2010).

[3] Valiant, L. G. The complexity of enumeration and reliability problems. *siam Journal on Computing* **8**, 410–421 (1979).

[4] Roberts, B. & Kroese, D. Estimating the number of st paths in a graph. *Journal of Graph Algorithms and Applications* **11**, 195–214 (2007).

[5] Estrada, E. & Hatano, N. Communicability in complex networks. *Physical Review E—Statistical, Nonlinear, and Soft Matter Physics* **77**, 036111 (2008).

[6] Benzi, M. & Klymko, C. Total communicability as a centrality measure. *Journal of Complex Networks* **1**, 124–149 (2013).

[7] Estrada, E. & Hatano, N. Communicability graph and community structures in complex networks. *Applied Mathematics and Computation* **214**, 500–511 (2009).

[8] Estrada, E. The communicability distance in graphs. *Linear Algebra and its Applications* **436**, 4317–4328 (2012).

[9] De la Cruz Cabrera, O., Jin, J., Noschese, S. & Reichel, L. Communication in complex networks. *Applied Numerical Mathematics* **172**, 186–205 (2022).

[10] Broniatowski, D. A., Simons, J. R., Gu, J., Jamison, A. M. & Abroms, L. C. The efficacy of facebook's vaccine misinformation policies and architecture during the covid-19 pandemic. *Science Advances* **9**, eadh2132 (2023).

[11] Moses, J. *The Anatomy of Large Scale Systems* (2012).

[12] Csardi, G. & Nepusz, T. The igraph software package for complex network research. *InterJournal* **Complex Systems**, 1695 (2006).

[13] Watts, D. J. & Strogatz, S. H. Collective dynamics of 'small-world'networks. *nature* **393**, 440–442 (1998).

[14] Barabási, A.-L. Scale-free networks: A decade and beyond. *Science* **325**, 412–413, DOI: 10.1126/science.1173299 (2009).

[15] Barabási, A.-L. & Bonabeau, E. Scale-free networks. *Scientific american* **288**, 50–9 (2003).

[16] Broido, A. D. & Clauset, A. Scale-free networks are rare. *Nature communications* **10**, 1017 (2019).

[17] Alstott, J., Bullmore, E. & Plenz, D. powerlaw: a python package for analysis of heavy-tailed distributions. *PloS one* **9**, e85777 (2014).

[18] Krippendorff, K. Measuring the reliability of qualitative text analysis data. *Quality and quantity* **38**, 787–800 (2004).
